# Crystal structures of three complexes of zinc chloride with tri-*tert*-butyl­phosphane

**DOI:** 10.1107/S2056989015023373

**Published:** 2016-01-01

**Authors:** Aaron D. Finke, Danielle L. Gray, Jeffrey S. Moore

**Affiliations:** a Swiss Light Source, Paul Scherrer Institute, 5232 Villigen PSI, Switzerland; b University of Illinois, School of Chemical Sciences, Box 59-1, 505 South Mathews Avenue, Urbana, Illinois 61801, USA; c University of Illinois, School of Chemical Sciences, Box 55-5, 600 South Mathews Avenue, Urbana, Illinois 61801, USA

**Keywords:** crystal structure, zinc chloride, tri-*tert*-butyl­phosphane, Lewis base

## Abstract

Under anhydrous conditions and in the absence of a Lewis-base solvent, a zinc chloride complex with tri-*tert*-butyl­phosphane as the μ-bridged dimer is formed, *viz.* di-μ-chlorido-bis­[chlorido­bis­(tri-*tert*-butyl­phosphane)zinc], [ZnCl_4_(C_12_H_27_P)_2_], (**1**), which features a nearly square-shaped (ZnCl)_2_ cyclic core and whose Cl atoms inter­act weakly with C—H groups on the phosphane ligand. In the presence of THF, monomeric di­chlorido­(tetra­hydro­furan-κ*O*)(tri-*tert*-butyl­phosphane-κ*P*)zinc, [ZnCl_2_(C_4_H_8_O)(C_12_H_27_P)] or [P(*t*Bu_3_)(THF)ZnCl_2_], (**2**), is formed. This slightly distorted tetra­hedral Zn complex has weak C—H⋯Cl inter­actions between the Cl atoms and phosphane and THF C—H groups. Under ambient conditions, the hydrolysed complex tri-*tert*-butyl­phospho­nium aqua­tri­chlorido­zincate 1,2-di­chloro­ethane monosolvate, (C_12_H_28_P)[ZnCl_3_(H_2_O)]·C_2_H_4_Cl_2_ or [HP*t*Bu_3_]^+^ [(H_2_O)ZnCl_3_]^−^·C_2_H_4_Cl_2_, (**3**), is formed. This complex forms chains of [(H_2_O)ZnCl_3_]^−^ anions from hydrogen-bonding inter­actions between the water H atoms and Cl atoms that propagate along the *b* axis.

## Abstract 2

The crystal structures of three complexes of zinc chloride with tri-*tert*-butyl­phosphane are reported.

## Chemical context   

Tri-*tert*-butyl­phosphane P*t*Bu_3_ is a bulky, weak Lewis base. It has found considerable utility as a ligand for Pd-catalysed cross couplings (Fu, 2008 ▸). More recently, its reactivity with bulky Lewis acids to form the so-called ‘frustrated Lewis pairs’ has opened up new avenues of chemical reactivity (Stephan & Erker, 2015 ▸). Lewis acidic complexes containing zinc have been used as ring-opening polymerization catalysts (Wu *et al.*, 2006 ▸). The reactivity of P*t*Bu_3_ with weak transition metal Lewis acids has been less well explored. The reaction of P*t*Bu_3_ with ZnCl_2_ has been reported (Goel & Ogini, 1977 ▸), but without structural characterization. Therein, [(P*t*Bu_3_)(ZnCl_2_)] (**1**) was proposed to exist as the di-μ-chlorido-bridged dimer based on mol­ecular weight measurements. We describe the structure of two complexes of [(P*t*Bu_3_)(ZnCl_2_)]: the aforementioned μ-bridged dimer (**1**), and the monomeric THF complex (**2**). The complex is sensitive to ambient moisture, and hydrolyses to form the hydrolysis product [HP*t*Bu_3_]^+^ [(H_2_O)ZnCl_3_]^−^·C_2_H_4_Cl_2_ (**3**) under ambient conditions from a 1,2-dichloroethane solution. The related compound [HP*t*Bu_3_]^+^[(H_2_O)ZnI_3_]^−^ was reported from the preparation of P*t*Bu_3_ and ZnI_2_ in benzene under ambient conditions (Goel & Ogini, 1977 ▸), but no structural data were reported.

## Structural commentary   

**Figure 1:**
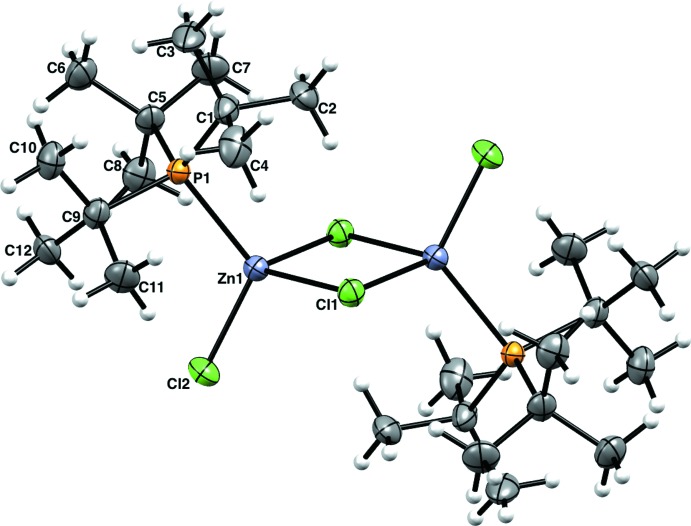
The molecular structure of (**1**), showing 50% probability ellipsoids for non-H atoms and spheres of arbitrary size for H atoms. The unlabeled atoms are related by the symmetry operator (−*x*, 1 − *y*, 1 − *z*).

Compound (**1**) is a neutral μ-bridged dimer with one P*t*Bu_3_ per zinc atom. The asymmetric unit is one half of (**1**) with the other half related by inversion symmetry (Fig. 1 ▸). The coordination sphere of the Zn is filled with two Cl atoms, one of which, Cl1, is μ-bound to both Zn atoms of (**1**) [Zn1—Cl1 = 2.3703 (13) Å] and the other, Cl2, is bound to only one Zn [Zn1—Cl2 = 2.2133 (14) Å]. The four-membered ring consisting of two Zn1 and two Cl1 is planar. The bond angles are only slightly distorted from the ideal values of 90° [Cl1—Zn1—Cl1^i^ = 90.98 (4), Zn1—Cl1—Zn1^i^ = 89.02 (4)°; symmetry code: (i) −*x*, −*y* + 1, −*z* + 1]. The Zn atom is a distorted tetra­hedron [P1—Zn1—Cl2 = 117.30 (5), P1—Zn1—Cl1 = 112.62 (5)°; τ_4_ = 0.92 (Yang *et al.*, 2007 ▸)]. The Zn⋯Zn^i^ distance is 3.3189 (10) Å [symmetry code: (i) −*x*, −*y* + 1, −*z* + 1). The Zn—P bond [Zn1—P1 = 2.3859 (13) Å] for (**1**) is in line with other Zn–tri­alkyl­phosphane complexes.
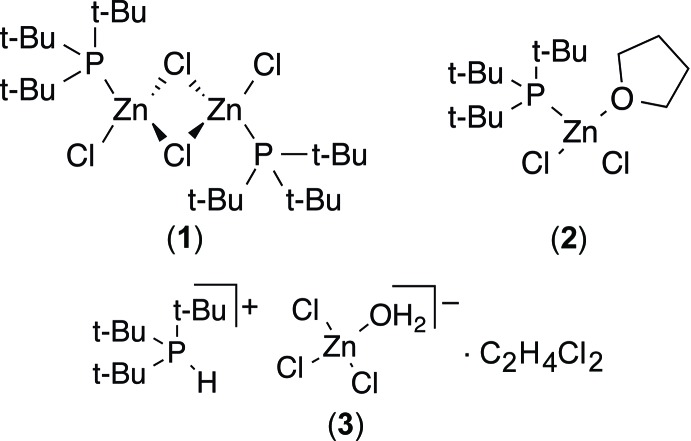


**Figure 2:**
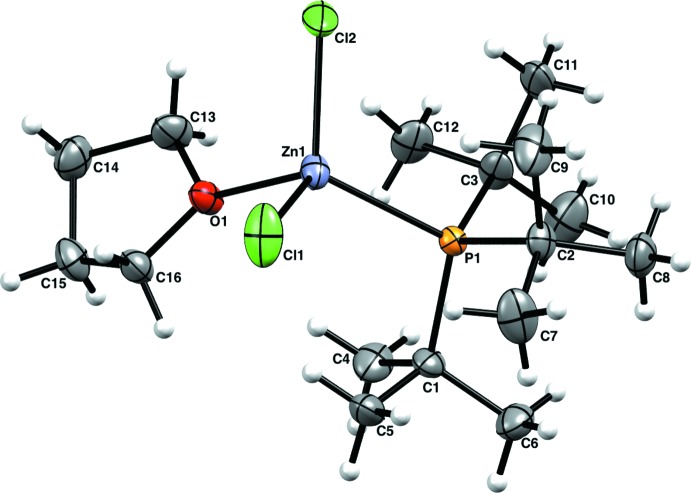
The molecular structure of (**2**), showing 50% probability ellipsoids for non-H atoms and spheres of arbitrary size for H atoms.

Compound (**2**) is a neutral tetra­hedral Zn complex with two Cl ligands, one P*t*Bu_3_ ligand, and one THF ligand (Fig. 2 ▸). The Zn—P bond length [Zn1—P1 = 2.4167 (9) Å] is in line with other Zn-tri­alkyl­phosphane complexes. The Zn—Cl bond lengths are very similar [Zn1—Cl1 = 2.2370 (13), Zn1—Cl2 = 2.2301 (13) Å]. The Zn environment is slightly distorted tetra­hedral due to the steric influence of the P*t*Bu_3_ ligand (τ_4_ = 0.94) (Yang *et al.*, 2007 ▸).

**Figure 3:**
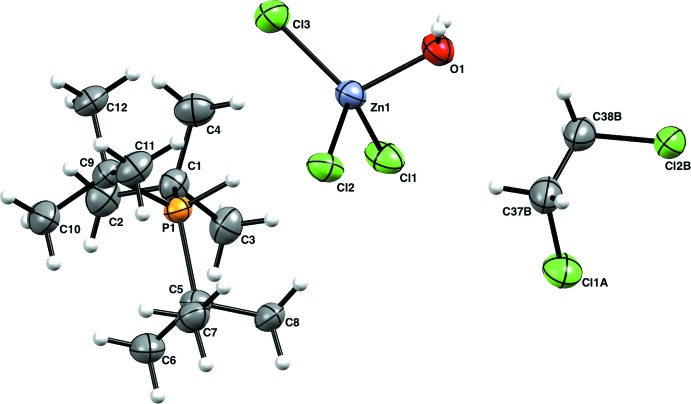
The molecular structure of (**3**), showing one of the three mol­ecules of the asymmetric unit (*Z*′ = 3) showing 50% probability ellipsoids for non-H atoms and spheres of arbitrary size for H atoms.

The asymmetric unit of compound (**3**) (Fig. 3 ▸) comprises three [HP*t*Bu_3_]^+^ [(H_2_O)ZnCl_3_]^−^ ion pairs, along with three 1,2-di­chloro­ethane mol­ecules not related by symmetry (*Z*′ = 3). The three groups are similar in structure, connectivity, and supra­molecular inter­actions; despite this, no additional crystallographic symmetry or twinning was found using *PLATON* (Spek, 2009 ▸). The [(H_2_O)ZnCl_3_]^−^ ion has some inter­esting properties. Two of the three Cl atoms in each [(H_2_O)ZnCl_3_]^−^ ion are involved in hydrogen bonding with nearby water ligands. The Zn—Cl bonds [Zn1—Cl1 = 2.2690 (10), Zn1—Cl2 = 2.2666 (10), Zn1—Cl3 = 2.2219 (11), Zn2—Cl4 = 2.2203 (11) Å, Zn2—Cl5 = 2.2666 (10) Å, Zn2—Cl6 = 2.2699 (10), Zn3—Cl7 = 2.2199 (11) Å, Zn3—Cl8 = 2.2695 (10), Zn3—Cl9 = 2.2686 (10) Å] are affected significantly by the hydrogen bonding. The Zn—Cl bonds involved in hydrogen bonding are significantly longer (by *ca* 0.04 Å) than the Zn—Cl bonds not involved in hydrogen bonding. The Zn—OH_2_ bonds [Zn1—O1 = 2.024 (3), Zn2—O2 = 2.025 (3), Zn3—O3 = 2.028 (3) Å] are all within one s.u. of the average tetra­hedral Zn—OH_2_ bond length of 2.00 (4) Å. The coordination environments of the Zn atoms in the [(H_2_O)ZnCl_3_]^−^ anions are all slightly distorted tetra­hedral [τ_4_(Zn1) = 0.92, τ_4_(Zn2) = 0.93, τ_4_(Zn3) = 0.93] (Yang *et al.*, 2007 ▸). The phospho­nium hydrogen atoms were found in a difference map and restrained to be similar to each other; the average P—H bond length is 1.31 (3) Å. The 1,2-di­chloro­ethane solvent has significantly larger displacement parameters than the other two moieties, indicating disorder. Thus, each solvent mol­ecule was modeled over two discrete positions (see *Refinement* section).

## Supra­molecular features   

**Figure 4:**
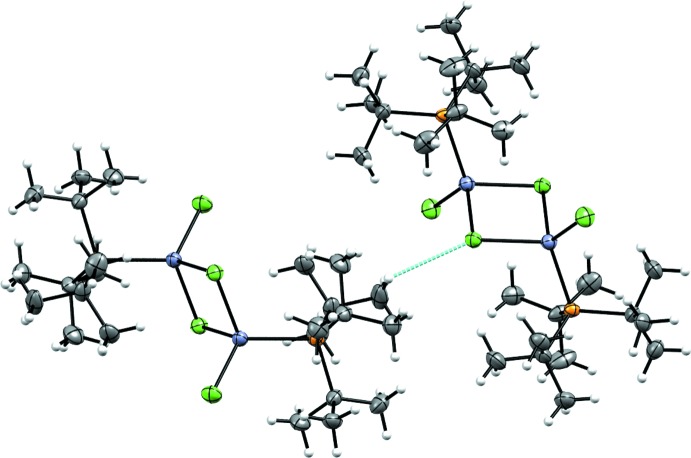
The weak H⋯Cl inter­action in (**1**) with short contact shown in cyan. The second mol­ecule (left) is related to the first by the symmetry operation (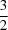 − *x*, −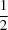 + *y*, 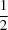 + *z*).

Supra­molecular features of (**1**) form from weak C1—H3*C*⋯Cl1^i^ inter­actions (Fig. 4 ▸ and Table 1 ▸), which creates layers in the *ab* plane that stack along the *c* axis.

**Figure 5:**
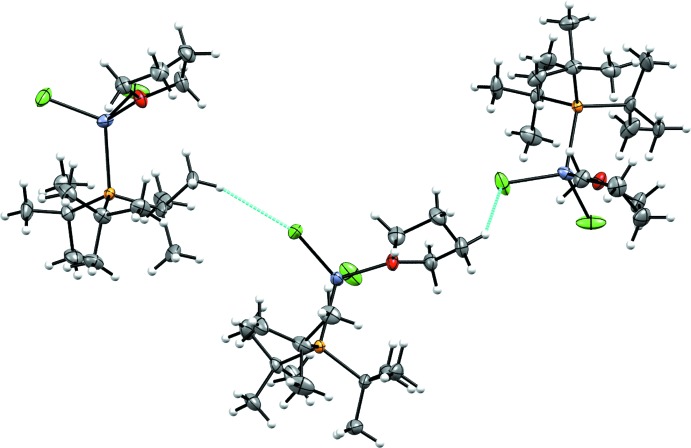
The weak H⋯Cl inter­actions in (**2**) with short contacts shown in cyan. The left mol­ecule is related to the middle one by the symmetry operation (2 − *x*, −*y*, 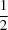 + *z*), and the right mol­ecule is related to the middle one by the symmetry operation (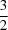 − *x*, −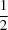 + *y*, 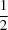 + *z*).

The supra­molecular features of (**2**) are also based on weak inter­actions. There are weak C15—H15*A*⋯Cl1^ii^ inter­actions as well as weak C12—H12*A*⋯Cl2^1^ inter­actions (Fig. 5 ▸ and Table 2 ▸). Together the weak inter­actions, where each Cl atom is an acceptor, create a three-dimensional packing structure.

**Figure 6:**
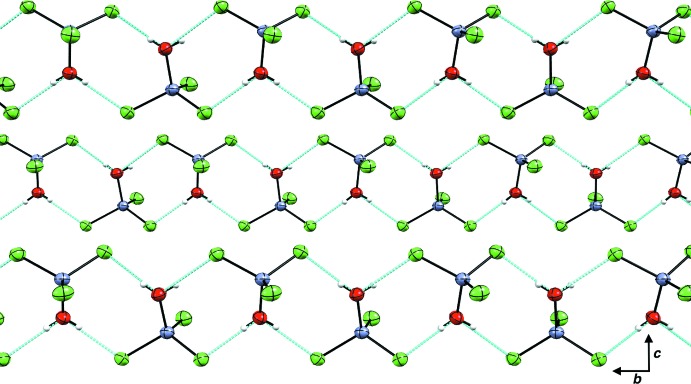
Chains of the three [(H_2_O)ZnCl_3_]^−^ ions formed from the hydrogen bonds between Zn—Cl and water ligands in (**3**), viewed along the *a* axis, with hydrogen-bond inter­actions shown in cyan. [HP*t*Bu_3_]^+^ ions and 1,2-DCE solvent mol­ecules are not shown.

The hydrogen atoms of the water ligands in (**3**) undergo hydrogen-bonding inter­actions with nearby chloride ligands of the [(H_2_O)ZnCl_3_]^−^ anion, forming chains that propagate along the *b*-axis direction (Fig. 6 ▸ and Table 3 ▸). The chains in each layer are staggered by half a unit cell along the *b* axis. The orientation of the P—H bond relative to the [(H_2_O)ZnCl_3_]^−^ ion is optimized for steric inter­actions; that is, the P—H hydrogen atom is oriented toward the center of the Zn tetra­hedron surrounded by three Cl atoms, suggesting a nucleophilic-type protonation of the phosphane, with the water ligand pointing away from the P—H bond. Each *tert*-butyl group is staggered slightly relative to the positions of the Cl atoms. In this arrangement, there are no hydrogen-bonding inter­actions involving the phospho­nium hydrogen. This arrangement also optimizes the ion contact between the phospho­nium cations and [(H_2_O)ZnCl_3_]^−^ anions. The disorder of the solvent mol­ecules suggests no or at best weak inter­actions between the solvent and hosts; indeed, none can be found.

## Database survey   

Zinc chloride-bulky phosphane compounds tend to form ZnCl_2_-monophosphane complexes. Two closely related compounds include a ZnCl_2_-phosphane dimer (LUZVEI; Liang *et al.*, 2010 ▸), and a ZnCl_2_-bulky NHC dimer (XONKUI; Fliedel *et al.*, 2014 ▸). In both cases, the mol­ecular geometries are very similar to that of (**1**). A search of the Cambridge Structural Database (CSD; Groom & Allen, 2014 ▸) returned 110 dimeric complexes with the general formula [*TM*(P*R*_3_Cl(μ-Cl)]_2_ (*TM* = transition metal). Most of these entries are complexes of group 10 metals (Ni, Pd, Pt), but, due to their different electron configuration to Zn, these tend to be mostly planar complexes. As expected, another Group 11 transition metal, Hg, forms similar complexes as Zn; there are 14 entries in the CSD with the formula [Hg(P*R*_3_Cl(μ-Cl)]_2_. Notably similar complexes to (**1**) include [Hg(P(cyclo­hex­yl)_3_Cl(μ-Cl)]_2_ (BULSOQ; Bell *et al.*, 1983 ▸) and [Hg(P(2,5-(OMe)_2_Ph)_3_Cl(μ-Cl)]_2_ (WONKEP; Bell *et al.*, 2000 ▸). Inter­estingly, there are no similar entries in the CSD that contain Cd.

There are three compounds in the CSD with the general formula [(thf)*TM*Cl_2_]. There is a compound closely related to (**2**), [ZnCl_2_(THF)(P(SnMe_3_)_3_)] (ASEBUV; Fuhr & Fenske, 2004 ▸). Like (**2**), it forms from the reaction of ZnCl_2_ with P(SnMe_3_)_3_ in THF. The other two compounds are complexes of Pd (FIRDAN, Cohen *et al.*, 2014 ▸; UHUDAC, Kim & Verkade, 2003 ▸).

Besides Goel’s report on the hydrolysis of [(P*t*Bu_3_)(ZnI_2_)], there are no other reports on the hydrolysis of zinc-phosphane complexes to form phospho­nium salts. The [(H_2_O)ZnCl_3_]^−^ ion is relatively uncommon in the CSD: there are 19 entries containing such an ion. However, there is one report of the hydrolysis of a tri­phenyl­phosphinomethyl–ZnCl_2_ dimer (CORRAD; Pattacini *et al.*, 2009 ▸) with water to form [Ph_3_PMe]^+^ [(H_2_O)ZnCl_3_]^−^ (CORQEG; Pattacini *et al.*, 2009 ▸). The [(H_2_O)ZnCl_3_]^−^ ions also form chains similar to (**3**) arising from hydrogen-bonding inter­actions between the two H atoms of the water ligand with two of the three Cl atoms of the ion. Likewise, the lengthening of the Zn—Cl bond as a result of hydrogen bonding as seen in (**3**) is also observed here. There are 67 entries in the CSD containing the moiety [HP*t*Bu_3_]^+^, none with Zn-containing counter-ions. Most of the counter-ions of [HP*t*Bu_3_]^+^ reported therein are anionic tetra­hedral borates arising from frustrated Lewis pair reactivity.

## Synthesis and crystallization   

The synthesis of (**1**) has been reported (Goel & Ogini, 1977 ▸); the methods reported here are modified from the original report. Crystals of (**1**) were grown from slow diffusion of pentane into an equimolar solution of ZnCl_2_ and P*t*Bu_3_ in (CH_2_Cl)_2_ at 243 K under an atmosphere of Ar gas. Crystals of (**2**) were grown from slow diffusion of pentane into an equimolar solution of ZnCl_2_ and P*t*Bu_3_ in THF at 243 K under an atmosphere of Ar gas. Crystals of (**3**) were grown from slow diffusion of pentane into an equimolar solution of ZnCl_2_ and P*t*Bu_3_ in 1,2-di­chloro­ethane (1,2-DCE) at room temperature under ambient conditions.

## Refinement   

Compound (**1**): A structural model consisting of one-half of (**1**) was developed. Methyl H atom positions, *R*—CH_3_, were optimized by rotation about *R–*-C bonds with idealized C—H, *R*—H and H⋯H distances. For all H atoms, *U*_iso_(H) = 1.5*U*_eq_(carrier).

Compound (**2**): A structural model consisting of the host mol­ecule was developed. The coordinating Cl atoms had elongated anisotropic displacement parameters in one direction; however, splitting the Cl positions did not significantly improve the model so it was removed from the final model. Methyl H atom positions, *R*—CH_3_, were optimized by rotation about *R*—C bonds with idealized C—H, *R*—H and H⋯H distances. Remaining H atoms were included as riding idealized contributors. *U*_iso_(H) = 1.5*U*_eq_(C) for methyl atoms and 1.2*U*_eq_(carrier) for remaining H atoms. On the basis of 1704 unmerged Friedel opposites, the minor component occupancy of the inversion twin was 0.206 (13) (Flack & Bernardinelli, 2000 ▸).

Compound (**3**): A structural model consisting of three ion pairs and three 1,2-DCE solvent mol­ecules per asymmetric unit was developed. Methyl H atom positions, *R–*-CH_3_, were optimized by rotation about *R*—C bonds with idealized C—H, *R*—H and H⋯H distances. Water H atoms and phospho­nium H atoms were identified in a difference Fourier map and refined. Water atom H atoms were restrained (s.u. 0.02) to a bond length of 0.84 Å. Phospho­nium H atoms were restrained to be similar (s.u. 0.01). Remaining H atoms were included as riding idealized contributors. *U*_iso_(H) = 1.5*U*_eq_(C) for methyl atoms and 1.2*U*_eq_(carrier) for remaining H atoms. The 1,2-DCE mol­ecules had significantly larger displacement parameters; thus, these moieties were modeled as disordered over two discrete positions. Enhanced rigid-bond restraints (s.u. 0.004) (Thorn *et al.*, 2012 ▸) were imposed on displacement parameters for all disordered sites and similar displacement amplitudes (s.u. 0.01) were imposed on disordered sites overlapping by less than the sum of van der Waals radii. In addition, the C—Cl bonds in the 1,2-DCE mol­ecules and the C—C bonds were restrained to be similar (s.u. 0.01). The major:minor occupancy factor ratios for the three 1,2-DCE mol­ecules are 0.52 (3):0.48 (3), 0.119 (7):0.881 (7), and 0.38 (3):0.62 (3). Crystal data, data collection and structure refinement details are summarized in Table 4 ▸.

## Supplementary Material

Crystal structure: contains datablock(s) 1, 2, 3. DOI: 10.1107/S2056989015023373/pk2569sup1.cif

Structure factors: contains datablock(s) 1. DOI: 10.1107/S2056989015023373/pk25691sup2.hkl

Supporting information file. DOI: 10.1107/S2056989015023373/pk25691sup5.cdx

Structure factors: contains datablock(s) 2. DOI: 10.1107/S2056989015023373/pk25692sup3.hkl

Supporting information file. DOI: 10.1107/S2056989015023373/pk25692sup6.cdx

Structure factors: contains datablock(s) 3. DOI: 10.1107/S2056989015023373/pk25693sup4.hkl

Supporting information file. DOI: 10.1107/S2056989015023373/pk25693sup7.cdx

CCDC references: 1440787, 1440786, 1440785

Additional supporting information:  crystallographic information; 3D view; checkCIF report

## Figures and Tables

**Table 1:** Hydrogen-bond geometry (Å, °) for (**1**)

*D*—H⋯*A*	*D*—H	H⋯*A*	*D*⋯*A*	*D*—H⋯*A*
C3—H3*C*⋯Cl1^i^	0.98	2.88	3.479 (6)	120

Symmetry code: (i) 
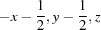
.

**Table 2:** Hydrogen-bond geometry (Å, °) for (**2**)

*D*—H⋯*A*	*D*—H	H⋯*A*	*D*⋯*A*	*D*—H⋯*A*
C12—H12*A*⋯Cl2^i^	0.98	2.90	3.819 (5)	157
C15—H15*A*⋯Cl1^ii^	0.99	2.94	3.747 (5)	140

Symmetry codes: (i) 
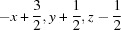
; (ii) 
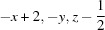
.

**Table 3:** Hydrogen-bond geometry (Å, °) for (**3**)

*D*—H⋯*A*	*D*—H	H⋯*A*	*D*⋯*A*	*D*—H⋯*A*
O1—H1*OA*⋯Cl2^i^	0.82 (2)	2.37 (3)	3.107 (3)	150 (4)
O1—H1*OB*⋯Cl1^ii^	0.82 (2)	2.27 (2)	3.086 (3)	173 (4)
O2—H2*OA*⋯Cl9^iii^	0.82 (2)	2.33 (2)	3.120 (3)	161 (4)
O2—H2*OB*⋯Cl8^iv^	0.82 (2)	2.28 (2)	3.095 (3)	177 (4)
O3—H3*OA*⋯Cl5^iii^	0.85 (2)	2.30 (2)	3.100 (3)	158 (4)
O3—H3*OB*⋯Cl6^iv^	0.83 (2)	2.28 (2)	3.106 (3)	173 (4)

Symmetry codes: (i) 
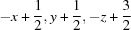
; (ii) 
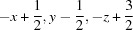
; (iii) 
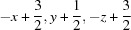
; (iv) 
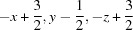
.

**Table 4:** Experimental details

	(**1**)	(**2**)	(**3**)
Crystal data			
Chemical formula	[ZnCl_4_(C_12_H_27_P)_2_]	[ZnCl_2_(C_4_H_8_O)(C_12_H_27_P)]	(C_12_H_28_P)[ZnCl_3_(H_2_O)]·C_2_H_4_Cl_2_
*M*_r_	677.15	410.68	492.00
Crystal system, space group	Orthorhombic, *P* *b* *c* *a*	Orthorhombic, *P* *n* *a*2_1_	Monoclinic, *P*2_1_/*n*
Temperature (K)	193	193	123
*a*, *b*, *c* (Å)	14.6408 (16), 12.9891 (14), 16.8190 (18)	26.4580 (11), 8.9281 (4), 8.5790 (4)	25.3722 (5), 8.5841 (2), 32.912 (2)
α, β, γ (°)	90, 90, 90	90, 90, 90	90, 98.909 (7), 90
*V* (Å^3^)	3198.5 (6)	2026.53 (16)	7081.7 (6)
*Z*	4	4	12
Radiation type	Mo *K*α	Mo *K*α	Cu *K*α
μ (mm^−1^)	1.95	1.55	7.28
Crystal size (mm)	0.22 × 0.18 × 0.09	0.47 × 0.46 × 0.27	0.18 × 0.05 × 0.02

Data collection			
Diffractometer	Bruker APEXII CCD area detector	Bruker APEXII CCD area detector	Rigaku CCD area detector
Absorption correction	Integration (*SADABS*; Bruker, 2008 ▸)	Integration (*SADABS*; Bruker, 2008 ▸)	Multi-scan (*CrystalClear*; Rigaku, 2010 ▸)
*T*_min_, *T*_max_	0.846, 0.999	0.580, 0.754	0.354, 0.868
No. of measured, independent and observed [*I* > 2σ(*I*)] reflections	32092, 2942, 1836	23933, 3698, 3581	67698, 11604, 7131
*R*_int_	0.169	0.038	0.083
(sin θ/λ)_max_ (Å^−1^)	0.604	0.603	0.581

Refinement			
*R*[*F* ^2^ > 2σ(*F* ^2^)], *wR*(*F* ^2^), *S*	0.045, 0.099, 1.00	0.031, 0.075, 1.14	0.049, 0.142, 0.98
No. of reflections	2942	3698	11604
No. of parameters	154	200	760
No. of restraints	0	1	462
H-atom treatment	H-atom parameters constrained	H-atom parameters constrained	H atoms treated by a mixture of independent and constrained refinement
Δρ_max_, Δρ_min_ (e Å^−3^)	0.44, −0.42	0.69, −0.30	0.51, −0.70
Absolute structure	–	Refined as an inversion twin	–
Absolute structure parameter	–	0.206 (18)	–

Computer programs: *APEX2* (Bruker, 2004 ▸), *CrystalClear* (Rigaku, 2010 ▸), *SAINT* and *XPREP* (Bruker, 2005 ▸), *SADABS* (Bruker, 2008 ▸) and *TWINABS* (Bruker, 2008 ▸), *SHELXS97* (Sheldrick, 2008 ▸), *SHELXL2014* (Sheldrick, 2015 ▸) and *OLEX2* (Bourhis *et al.*, 2015 ▸).

## supplementary crystallographic information

### (1) Di-µ-chlorido-bis[chloridobis(tri-tert-butylphosphane)zinc] Crystal data

**Table:**

[ZnCl_4_(C_12_H_27_P)_2_]	*D*_x_ = 1.406 Mg m^−^^3^
*M**_r_* = 677.15	Mo *K*α radiation, λ = 0.71073 Å
Orthorhombic, *P**b**c**a*	Cell parameters from 1388 reflections
*a* = 14.6408 (16) Å	θ = 2.4–18.9°
*b* = 12.9891 (14) Å	µ = 1.95 mm^−^^1^
*c* = 16.8190 (18) Å	*T* = 193 K
*V* = 3198.5 (6) Å^3^	Block, colourless
*Z* = 4	0.22 × 0.18 × 0.09 mm
*F*(000) = 1424	

### (1) Di-µ-chlorido-bis[chloridobis(tri-tert-butylphosphane)zinc] Data collection

**Table:**

Bruker APEXII CCD area-detector diffractometer	2942 independent reflections
Radiation source: Sealed Tube	1836 reflections with *I* > 2σ(*I*)
Graphite monochromator	*R*_int_ = 0.169
Detector resolution: 8.33 pixels mm^-1^	θ_max_ = 25.4°, θ_min_ = 2.4°
φ and ω scans	*h* = −16→17
Absorption correction: integration (SADABS; Bruker, 2008)	*k* = −15→15
*T*_min_ = 0.846, *T*_max_ = 0.999	*l* = −20→20
32092 measured reflections	

### (1) Di-µ-chlorido-bis[chloridobis(tri-tert-butylphosphane)zinc] Refinement

**Table:**

Refinement on *F*^2^	0 restraints
Least-squares matrix: full	Hydrogen site location: inferred from neighbouring sites
*R*[*F*^2^ > 2σ(*F*^2^)] = 0.045	H-atom parameters constrained
*wR*(*F*^2^) = 0.099	*w* = 1/[σ^2^(*F*_o_^2^) + (0.0282*P*)^2^ + 3.8822*P*] where *P* = (*F*_o_^2^ + 2*F*_c_^2^)/3
*S* = 1.00	(Δ/σ)_max_ < 0.001
2942 reflections	Δρ_max_ = 0.44 e Å^−^^3^
154 parameters	Δρ_min_ = −0.42 e Å^−^^3^

### (1) Di-µ-chlorido-bis[chloridobis(tri-tert-butylphosphane)zinc] Special details

**Table:**

Geometry. All e.s.d.'s (except the e.s.d. in the dihedral angle between two l.s. planes) are estimated using the full covariance matrix. The cell e.s.d.'s are taken into account individually in the estimation of e.s.d.'s in distances, angles and torsion angles; correlations between e.s.d.'s in cell parameters are only used when they are defined by crystal symmetry. An approximate (isotropic) treatment of cell e.s.d.'s is used for estimating e.s.d.'s involving l.s. planes.

### (1) Di-µ-chlorido-bis[chloridobis(tri-tert-butylphosphane)zinc] Fractional atomic coordinates and isotropic or equivalent isotropic displacement parameters (Å^2^)

**Table:**

	*x*	*y*	*z*	*U*_iso_*/*U*_eq_	
Zn1	0.01390 (4)	0.41919 (4)	0.42454 (3)	0.02543 (17)	
Cl1	−0.07300 (8)	0.57038 (9)	0.44450 (7)	0.0287 (3)	
Cl2	0.12470 (9)	0.45042 (10)	0.33832 (8)	0.0372 (3)	
P1	−0.07908 (9)	0.27135 (9)	0.40137 (7)	0.0229 (3)	
C1	−0.1263 (4)	0.2197 (4)	0.4984 (3)	0.0340 (13)	
C2	−0.1597 (4)	0.3112 (4)	0.5491 (3)	0.0412 (14)	
H2A	−0.1840	0.2855	0.5997	0.062*	
H2B	−0.2078	0.3482	0.5204	0.062*	
H2C	−0.1086	0.3579	0.5595	0.062*	
C3	−0.2037 (4)	0.1424 (4)	0.4882 (3)	0.0505 (17)	
H3A	−0.2239	0.1183	0.5405	0.076*	
H3B	−0.1821	0.0837	0.4568	0.076*	
H3C	−0.2549	0.1754	0.4606	0.076*	
C4	−0.0504 (4)	0.1693 (4)	0.5459 (3)	0.0464 (16)	
H4A	−0.0733	0.1509	0.5988	0.070*	
H4B	0.0008	0.2175	0.5513	0.070*	
H4C	−0.0297	0.1071	0.5184	0.070*	
C5	−0.1755 (3)	0.3087 (4)	0.3323 (3)	0.0322 (13)	
C6	−0.2210 (4)	0.2184 (4)	0.2886 (3)	0.0429 (15)	
H6A	−0.2687	0.2448	0.2532	0.064*	
H6B	−0.2483	0.1713	0.3275	0.064*	
H6C	−0.1750	0.1815	0.2573	0.064*	
C7	−0.2506 (4)	0.3675 (4)	0.3768 (3)	0.0457 (15)	
H7A	−0.2951	0.3944	0.3386	0.069*	
H7B	−0.2233	0.4248	0.4064	0.069*	
H7C	−0.2814	0.3209	0.4140	0.069*	
C8	−0.1373 (4)	0.3861 (4)	0.2711 (3)	0.0390 (14)	
H8A	−0.1847	0.4026	0.2320	0.059*	
H8B	−0.0845	0.3557	0.2440	0.059*	
H8C	−0.1184	0.4492	0.2985	0.059*	
C9	−0.0043 (3)	0.1705 (3)	0.3523 (3)	0.0301 (12)	
C10	−0.0439 (4)	0.0616 (4)	0.3532 (3)	0.0405 (14)	
H10A	−0.0025	0.0150	0.3250	0.061*	
H10B	−0.1036	0.0617	0.3268	0.061*	
H10C	−0.0510	0.0385	0.4083	0.061*	
C11	0.0904 (3)	0.1705 (4)	0.3942 (3)	0.0424 (14)	
H11A	0.1301	0.1193	0.3690	0.064*	
H11B	0.0825	0.1533	0.4505	0.064*	
H11C	0.1181	0.2389	0.3895	0.064*	
C12	0.0166 (4)	0.2009 (4)	0.2653 (3)	0.0393 (14)	
H12A	0.0631	0.1546	0.2436	0.059*	
H12B	0.0392	0.2719	0.2637	0.059*	
H12C	−0.0393	0.1956	0.2335	0.059*	

### (1) Di-µ-chlorido-bis[chloridobis(tri-tert-butylphosphane)zinc] Atomic displacement parameters (Å^2^)

**Table:**

	*U*^11^	*U*^22^	*U*^33^	*U*^12^	*U*^13^	*U*^23^
Zn1	0.0231 (3)	0.0292 (3)	0.0240 (3)	−0.0022 (3)	0.0020 (3)	−0.0028 (2)
Cl1	0.0262 (7)	0.0331 (6)	0.0267 (7)	0.0034 (6)	−0.0036 (5)	−0.0036 (5)
Cl2	0.0343 (8)	0.0456 (8)	0.0318 (8)	−0.0039 (6)	0.0110 (6)	−0.0018 (6)
P1	0.0229 (7)	0.0272 (7)	0.0185 (7)	−0.0020 (6)	−0.0010 (6)	−0.0017 (5)
C1	0.043 (3)	0.039 (3)	0.020 (3)	−0.013 (3)	0.004 (3)	−0.004 (2)
C2	0.038 (3)	0.059 (4)	0.027 (3)	−0.011 (3)	0.010 (3)	−0.007 (3)
C3	0.060 (4)	0.054 (4)	0.038 (4)	−0.032 (3)	0.017 (3)	−0.005 (3)
C4	0.070 (4)	0.043 (3)	0.026 (3)	−0.004 (3)	−0.004 (3)	0.008 (2)
C5	0.024 (3)	0.039 (3)	0.034 (3)	0.006 (2)	−0.008 (3)	−0.007 (2)
C6	0.035 (3)	0.054 (4)	0.040 (3)	−0.001 (3)	−0.009 (3)	−0.012 (3)
C7	0.028 (3)	0.058 (4)	0.051 (4)	0.007 (3)	−0.009 (3)	−0.005 (3)
C8	0.042 (4)	0.044 (3)	0.032 (3)	0.003 (3)	−0.010 (3)	0.004 (2)
C9	0.031 (3)	0.027 (2)	0.032 (3)	0.003 (2)	−0.005 (3)	−0.006 (2)
C10	0.049 (4)	0.032 (3)	0.040 (3)	0.005 (3)	−0.004 (3)	−0.008 (2)
C11	0.028 (3)	0.047 (3)	0.052 (4)	0.010 (3)	−0.006 (3)	−0.006 (3)
C12	0.029 (3)	0.054 (3)	0.035 (3)	0.003 (3)	0.005 (3)	−0.015 (3)

### (1) Di-µ-chlorido-bis[chloridobis(tri-tert-butylphosphane)zinc] Geometric parameters (Å, º)

**Table:**

Zn1—Cl1^i^	2.3703 (13)	C5—C8	1.544 (7)
Zn1—Cl1	2.3639 (13)	C6—H6A	0.9800
Zn1—Cl2	2.2133 (14)	C6—H6B	0.9800
Zn1—P1	2.3859 (13)	C6—H6C	0.9800
Cl1—Zn1^i^	2.3704 (13)	C7—H7A	0.9800
P1—C1	1.894 (5)	C7—H7B	0.9800
P1—C5	1.891 (5)	C7—H7C	0.9800
P1—C9	1.896 (5)	C8—H8A	0.9800
C1—C2	1.543 (7)	C8—H8B	0.9800
C1—C3	1.524 (7)	C8—H8C	0.9800
C1—C4	1.517 (7)	C9—C10	1.528 (6)
C2—H2A	0.9800	C9—C11	1.555 (7)
C2—H2B	0.9800	C9—C12	1.546 (7)
C2—H2C	0.9800	C10—H10A	0.9800
C3—H3A	0.9800	C10—H10B	0.9800
C3—H3B	0.9800	C10—H10C	0.9800
C3—H3C	0.9800	C11—H11A	0.9800
C4—H4A	0.9800	C11—H11B	0.9800
C4—H4B	0.9800	C11—H11C	0.9800
C4—H4C	0.9800	C12—H12A	0.9800
C5—C6	1.536 (7)	C12—H12B	0.9800
C5—C7	1.534 (7)	C12—H12C	0.9800
			
Cl1—Zn1—Cl1^i^	90.98 (4)	C5—C6—H6A	109.5
Cl1—Zn1—P1	112.62 (5)	C5—C6—H6B	109.5
Cl1^i^—Zn1—P1	113.96 (5)	C5—C6—H6C	109.5
Cl2—Zn1—Cl1^i^	109.32 (5)	H6A—C6—H6B	109.5
Cl2—Zn1—Cl1	109.59 (5)	H6A—C6—H6C	109.5
Cl2—Zn1—P1	117.30 (5)	H6B—C6—H6C	109.5
Zn1—Cl1—Zn1^i^	89.02 (4)	C5—C7—H7A	109.5
C1—P1—Zn1	110.64 (16)	C5—C7—H7B	109.5
C1—P1—C9	109.9 (2)	C5—C7—H7C	109.5
C5—P1—Zn1	108.67 (16)	H7A—C7—H7B	109.5
C5—P1—C1	110.3 (2)	H7A—C7—H7C	109.5
C5—P1—C9	109.9 (2)	H7B—C7—H7C	109.5
C9—P1—Zn1	107.32 (16)	C5—C8—H8A	109.5
C2—C1—P1	108.6 (3)	C5—C8—H8B	109.5
C3—C1—P1	114.1 (4)	C5—C8—H8C	109.5
C3—C1—C2	109.5 (4)	H8A—C8—H8B	109.5
C4—C1—P1	109.9 (4)	H8A—C8—H8C	109.5
C4—C1—C2	105.8 (4)	H8B—C8—H8C	109.5
C4—C1—C3	108.7 (4)	C10—C9—P1	114.6 (4)
C1—C2—H2A	109.5	C10—C9—C11	109.5 (4)
C1—C2—H2B	109.5	C10—C9—C12	108.7 (4)
C1—C2—H2C	109.5	C11—C9—P1	108.5 (3)
H2A—C2—H2B	109.5	C12—C9—P1	110.5 (3)
H2A—C2—H2C	109.5	C12—C9—C11	104.6 (4)
H2B—C2—H2C	109.5	C9—C10—H10A	109.5
C1—C3—H3A	109.5	C9—C10—H10B	109.5
C1—C3—H3B	109.5	C9—C10—H10C	109.5
C1—C3—H3C	109.5	H10A—C10—H10B	109.5
H3A—C3—H3B	109.5	H10A—C10—H10C	109.5
H3A—C3—H3C	109.5	H10B—C10—H10C	109.5
H3B—C3—H3C	109.5	C9—C11—H11A	109.5
C1—C4—H4A	109.5	C9—C11—H11B	109.5
C1—C4—H4B	109.5	C9—C11—H11C	109.5
C1—C4—H4C	109.5	H11A—C11—H11B	109.5
H4A—C4—H4B	109.5	H11A—C11—H11C	109.5
H4A—C4—H4C	109.5	H11B—C11—H11C	109.5
H4B—C4—H4C	109.5	C9—C12—H12A	109.5
C6—C5—P1	115.0 (3)	C9—C12—H12B	109.5
C6—C5—C8	109.6 (4)	C9—C12—H12C	109.5
C7—C5—P1	111.3 (4)	H12A—C12—H12B	109.5
C7—C5—C6	107.7 (4)	H12A—C12—H12C	109.5
C7—C5—C8	105.1 (4)	H12B—C12—H12C	109.5
C8—C5—P1	107.8 (3)		
			
Zn1—P1—C1—C2	43.7 (4)	C1—P1—C9—C12	167.1 (3)
Zn1—P1—C1—C3	166.1 (4)	C5—P1—C1—C2	−76.6 (4)
Zn1—P1—C1—C4	−71.7 (4)	C5—P1—C1—C3	45.8 (5)
Zn1—P1—C5—C6	159.6 (3)	C5—P1—C1—C4	168.0 (3)
Zn1—P1—C5—C7	−77.7 (4)	C5—P1—C9—C10	−77.7 (4)
Zn1—P1—C5—C8	37.0 (4)	C5—P1—C9—C11	159.6 (3)
Zn1—P1—C9—C10	164.3 (3)	C5—P1—C9—C12	45.5 (4)
Zn1—P1—C9—C11	41.6 (4)	C9—P1—C1—C2	162.0 (3)
Zn1—P1—C9—C12	−72.6 (3)	C9—P1—C1—C3	−75.6 (4)
C1—P1—C5—C6	−79.0 (4)	C9—P1—C1—C4	46.7 (4)
C1—P1—C5—C7	43.8 (4)	C9—P1—C5—C6	42.4 (4)
C1—P1—C5—C8	158.5 (3)	C9—P1—C5—C7	165.1 (3)
C1—P1—C9—C10	43.9 (4)	C9—P1—C5—C8	−80.1 (4)
C1—P1—C9—C11	−78.8 (4)		

Symmetry code: (i) −*x*, −*y*+1, −*z*+1.

### (1) Di-µ-chlorido-bis[chloridobis(tri-tert-butylphosphane)zinc] Hydrogen-bond geometry (Å, º)

**Table:**

*D*—H···*A*	*D*—H	H···*A*	*D*···*A*	*D*—H···*A*
C3—H3*C*···Cl1^ii^	0.98	2.88	3.479 (6)	120

Symmetry code: (ii) −*x*−1/2, *y*−1/2, *z*.

### (2) Dichlorido(tetrahydrofuran-κO)(tri-tert-butylphosphane-κP)zinc Crystal data

**Table:**

[ZnCl_2_(C_4_H_8_O)(C_12_H_27_P)]	*D*_x_ = 1.346 Mg m^−^^3^
*M**_r_* = 410.68	Mo *K*α radiation, λ = 0.71073 Å
Orthorhombic, *P**n**a*2_1_	Cell parameters from 9968 reflections
*a* = 26.4580 (11) Å	θ = 2.8–27.6°
*b* = 8.9281 (4) Å	µ = 1.55 mm^−^^1^
*c* = 8.5790 (4) Å	*T* = 193 K
*V* = 2026.53 (16) Å^3^	Block, colourless
*Z* = 4	0.47 × 0.46 × 0.27 mm
*F*(000) = 872	

### (2) Dichlorido(tetrahydrofuran-κO)(tri-tert-butylphosphane-κP)zinc Data collection

**Table:**

Bruker APEXII CCD area-detector diffractometer	3698 independent reflections
Radiation source: sealed tube	3581 reflections with *I* > 2σ(*I*)
Graphite monochromator	*R*_int_ = 0.038
Detector resolution: 8.33 pixels mm^-1^	θ_max_ = 25.4°, θ_min_ = 2.4°
ω and φ scans	*h* = −31→31
Absorption correction: integration (SADABS; Bruker, 2008)	*k* = −10→10
*T*_min_ = 0.580, *T*_max_ = 0.754	*l* = −10→10
23933 measured reflections	

### (2) Dichlorido(tetrahydrofuran-κO)(tri-tert-butylphosphane-κP)zinc Refinement

**Table:**

Refinement on *F*^2^	Hydrogen site location: inferred from neighbouring sites
Least-squares matrix: full	H-atom parameters constrained
*R*[*F*^2^ > 2σ(*F*^2^)] = 0.031	*w* = 1/[σ^2^(*F*_o_^2^) + (0.0318*P*)^2^ + 1.5653*P*] where *P* = (*F*_o_^2^ + 2*F*_c_^2^)/3
*wR*(*F*^2^) = 0.075	(Δ/σ)_max_ = 0.001
*S* = 1.14	Δρ_max_ = 0.69 e Å^−^^3^
3698 reflections	Δρ_min_ = −0.30 e Å^−^^3^
200 parameters	Absolute structure: Refined as an inversion twin.
1 restraint	Absolute structure parameter: 0.206 (18)

### (2) Dichlorido(tetrahydrofuran-κO)(tri-tert-butylphosphane-κP)zinc Special details

**Table:**

Experimental. One distinct cell was identified using *APEX2* (Bruker, 2004). Six frame series were integrated and filtered for statistical outliers using *SAINT* (Bruker, 2005) then corrected for absorption by integration using *SHELXTL*/*XPREP* V2005/2 (Bruker, 2005) before using *SAINT*/*SADABS* (Bruker, 2005) to sort, merge, and scale the combined data. No decay correction was applied.
Geometry. All e.s.d.'s (except the e.s.d. in the dihedral angle between two l.s. planes) are estimated using the full covariance matrix. The cell e.s.d.'s are taken into account individually in the estimation of e.s.d.'s in distances, angles and torsion angles; correlations between e.s.d.'s in cell parameters are only used when they are defined by crystal symmetry. An approximate (isotropic) treatment of cell e.s.d.'s is used for estimating e.s.d.'s involving l.s. planes.
Refinement. Refined as a 2-component inversion twin.

### (2) Dichlorido(tetrahydrofuran-κO)(tri-tert-butylphosphane-κP)zinc Fractional atomic coordinates and isotropic or equivalent isotropic displacement parameters (Å^2^)

**Table:**

	*x*	*y*	*z*	*U*_iso_*/*U*_eq_	
Zn1	0.88274 (2)	0.23668 (4)	0.83834 (8)	0.02419 (14)	
Cl1	0.95342 (5)	0.17357 (16)	0.96546 (16)	0.0477 (4)	
Cl2	0.81608 (5)	0.08915 (12)	0.88009 (17)	0.0494 (4)	
P1	0.86195 (3)	0.49987 (10)	0.85327 (14)	0.0168 (2)	
O1	0.90182 (12)	0.1814 (4)	0.6087 (4)	0.0266 (7)	
C1	0.91147 (16)	0.6144 (5)	0.7468 (5)	0.0257 (9)	
C2	0.96425 (16)	0.5523 (5)	0.7851 (6)	0.0342 (11)	
H2A	0.9897	0.6048	0.7224	0.051*	
H2B	0.9653	0.4450	0.7612	0.051*	
H2C	0.9714	0.5675	0.8960	0.051*	
C3	0.9103 (2)	0.7834 (5)	0.7839 (6)	0.0357 (12)	
H3A	0.9352	0.8355	0.7190	0.054*	
H3B	0.9185	0.7990	0.8941	0.054*	
H3C	0.8765	0.8232	0.7621	0.054*	
C4	0.9053 (2)	0.5948 (6)	0.5701 (6)	0.0369 (12)	
H4A	0.9325	0.6482	0.5161	0.055*	
H4B	0.8726	0.6358	0.5376	0.055*	
H4C	0.9068	0.4881	0.5439	0.055*	
C5	0.86075 (17)	0.5543 (5)	1.0665 (5)	0.0256 (9)	
C6	0.9152 (2)	0.5661 (7)	1.1274 (6)	0.0414 (15)	
H6A	0.9147	0.5778	1.2410	0.062*	
H6B	0.9317	0.6531	1.0801	0.062*	
H6C	0.9338	0.4750	1.1000	0.062*	
C7	0.8333 (2)	0.7020 (6)	1.1031 (7)	0.0370 (11)	
H7A	0.8381	0.7274	1.2132	0.055*	
H7B	0.7971	0.6905	1.0815	0.055*	
H7C	0.8471	0.7823	1.0378	0.055*	
C8	0.8363 (3)	0.4261 (6)	1.1571 (6)	0.0464 (14)	
H8A	0.8371	0.4488	1.2689	0.070*	
H8B	0.8550	0.3332	1.1374	0.070*	
H8C	0.8012	0.4140	1.1233	0.070*	
C9	0.79715 (16)	0.5310 (5)	0.7633 (6)	0.0292 (10)	
C10	0.7853 (2)	0.6937 (6)	0.7234 (8)	0.0479 (14)	
H10A	0.7507	0.7011	0.6841	0.072*	
H10B	0.8089	0.7289	0.6432	0.072*	
H10C	0.7889	0.7556	0.8170	0.072*	
C11	0.75568 (15)	0.4718 (6)	0.8714 (7)	0.0433 (14)	
H11A	0.7229	0.4780	0.8184	0.065*	
H11B	0.7547	0.5323	0.9667	0.065*	
H11C	0.7628	0.3672	0.8983	0.065*	
C12	0.79414 (19)	0.4325 (6)	0.6168 (6)	0.0356 (12)	
H12A	0.7611	0.4456	0.5672	0.053*	
H12B	0.7986	0.3272	0.6460	0.053*	
H12C	0.8208	0.4619	0.5437	0.053*	
C13	0.86717 (17)	0.1097 (6)	0.4985 (6)	0.0344 (11)	
H13A	0.8461	0.1856	0.4448	0.041*	
H13B	0.8446	0.0384	0.5530	0.041*	
C14	0.90056 (19)	0.0286 (6)	0.3834 (6)	0.0390 (12)	
H14A	0.8853	0.0281	0.2780	0.047*	
H14B	0.9069	−0.0759	0.4167	0.047*	
C15	0.94860 (18)	0.1199 (6)	0.3865 (6)	0.0360 (11)	
H15A	0.9779	0.0601	0.3505	0.043*	
H15B	0.9455	0.2107	0.3209	0.043*	
C16	0.95347 (17)	0.1599 (6)	0.5564 (6)	0.0299 (10)	
H16A	0.9700	0.0781	0.6153	0.036*	
H16B	0.9734	0.2528	0.5697	0.036*	

### (2) Dichlorido(tetrahydrofuran-κO)(tri-tert-butylphosphane-κP)zinc Atomic displacement parameters (Å^2^)

**Table:**

	*U*^11^	*U*^22^	*U*^33^	*U*^12^	*U*^13^	*U*^23^
Zn1	0.0309 (2)	0.0208 (2)	0.0208 (2)	0.00455 (17)	0.0058 (3)	0.0024 (3)
Cl1	0.0617 (8)	0.0524 (7)	0.0288 (6)	0.0304 (6)	−0.0150 (6)	−0.0030 (6)
Cl2	0.0564 (7)	0.0252 (5)	0.0666 (11)	−0.0062 (5)	0.0398 (7)	−0.0007 (6)
P1	0.0177 (4)	0.0191 (4)	0.0138 (5)	0.0009 (3)	−0.0005 (5)	0.0008 (5)
O1	0.0250 (15)	0.0374 (17)	0.0175 (15)	−0.0053 (13)	0.0032 (13)	−0.0063 (13)
C1	0.028 (2)	0.027 (2)	0.022 (2)	−0.0059 (18)	0.0090 (18)	−0.0005 (18)
C2	0.025 (2)	0.039 (3)	0.039 (3)	−0.0069 (19)	0.0081 (19)	−0.009 (2)
C3	0.046 (3)	0.027 (2)	0.035 (3)	−0.007 (2)	0.007 (2)	−0.0012 (19)
C4	0.055 (3)	0.033 (3)	0.023 (3)	−0.006 (2)	0.011 (2)	0.004 (2)
C5	0.032 (2)	0.030 (2)	0.014 (2)	0.0020 (19)	0.0022 (17)	−0.0040 (18)
C6	0.044 (3)	0.061 (4)	0.019 (3)	0.013 (3)	−0.012 (2)	−0.008 (2)
C7	0.042 (3)	0.035 (3)	0.034 (3)	0.005 (2)	0.004 (2)	−0.013 (2)
C8	0.076 (4)	0.043 (3)	0.020 (2)	0.005 (3)	0.015 (3)	0.003 (2)
C9	0.018 (2)	0.031 (2)	0.039 (3)	0.0066 (17)	−0.0133 (19)	−0.008 (2)
C10	0.049 (3)	0.038 (3)	0.056 (4)	0.016 (2)	−0.028 (3)	−0.003 (3)
C11	0.020 (2)	0.046 (3)	0.063 (4)	0.0013 (18)	0.002 (2)	−0.021 (3)
C12	0.032 (2)	0.040 (3)	0.036 (3)	0.003 (2)	−0.018 (2)	−0.007 (2)
C13	0.029 (2)	0.047 (3)	0.028 (3)	−0.008 (2)	−0.0043 (19)	−0.009 (2)
C14	0.049 (3)	0.039 (3)	0.029 (3)	−0.002 (2)	−0.001 (2)	−0.011 (2)
C15	0.037 (2)	0.046 (3)	0.025 (3)	0.004 (2)	0.0101 (19)	−0.004 (2)
C16	0.023 (2)	0.037 (2)	0.029 (3)	−0.0011 (19)	0.0047 (18)	−0.004 (2)

### (2) Dichlorido(tetrahydrofuran-κO)(tri-tert-butylphosphane-κP)zinc Geometric parameters (Å, º)

**Table:**

Zn1—Cl1	2.2370 (13)	C7—H7B	0.9800
Zn1—Cl2	2.2301 (13)	C7—H7C	0.9800
Zn1—P1	2.4167 (9)	C8—H8A	0.9800
Zn1—O1	2.093 (3)	C8—H8B	0.9800
P1—C1	1.896 (4)	C8—H8C	0.9800
P1—C5	1.893 (5)	C9—C10	1.525 (7)
P1—C9	1.900 (4)	C9—C11	1.531 (7)
O1—C13	1.464 (5)	C9—C12	1.536 (7)
O1—C16	1.451 (5)	C10—H10A	0.9800
C1—C2	1.538 (6)	C10—H10B	0.9800
C1—C3	1.543 (6)	C10—H10C	0.9800
C1—C4	1.535 (7)	C11—H11A	0.9800
C2—H2A	0.9800	C11—H11B	0.9800
C2—H2B	0.9800	C11—H11C	0.9800
C2—H2C	0.9800	C12—H12A	0.9800
C3—H3A	0.9800	C12—H12B	0.9800
C3—H3B	0.9800	C12—H12C	0.9800
C3—H3C	0.9800	C13—H13A	0.9900
C4—H4A	0.9800	C13—H13B	0.9900
C4—H4B	0.9800	C13—C14	1.510 (7)
C4—H4C	0.9800	C14—H14A	0.9900
C5—C6	1.535 (7)	C14—H14B	0.9900
C5—C7	1.538 (6)	C14—C15	1.510 (7)
C5—C8	1.528 (7)	C15—H15A	0.9900
C6—H6A	0.9800	C15—H15B	0.9900
C6—H6B	0.9800	C15—C16	1.507 (6)
C6—H6C	0.9800	C16—H16A	0.9900
C7—H7A	0.9800	C16—H16B	0.9900
			
Cl1—Zn1—P1	114.16 (5)	H7B—C7—H7C	109.5
Cl2—Zn1—Cl1	115.72 (6)	C5—C8—H8A	109.5
Cl2—Zn1—P1	112.69 (4)	C5—C8—H8B	109.5
O1—Zn1—Cl1	101.41 (10)	C5—C8—H8C	109.5
O1—Zn1—Cl2	101.70 (9)	H8A—C8—H8B	109.5
O1—Zn1—P1	109.51 (10)	H8A—C8—H8C	109.5
C1—P1—Zn1	109.97 (14)	H8B—C8—H8C	109.5
C1—P1—C9	110.4 (2)	C10—C9—P1	114.5 (3)
C5—P1—Zn1	107.75 (15)	C10—C9—C11	108.6 (4)
C5—P1—C1	109.8 (2)	C10—C9—C12	110.5 (4)
C5—P1—C9	109.9 (2)	C11—C9—P1	110.5 (3)
C9—P1—Zn1	109.02 (14)	C11—C9—C12	105.1 (4)
C13—O1—Zn1	124.0 (3)	C12—C9—P1	107.2 (3)
C16—O1—Zn1	123.3 (3)	C9—C10—H10A	109.5
C16—O1—C13	109.4 (3)	C9—C10—H10B	109.5
C2—C1—P1	109.3 (3)	C9—C10—H10C	109.5
C2—C1—C3	109.1 (4)	H10A—C10—H10B	109.5
C3—C1—P1	114.5 (3)	H10A—C10—H10C	109.5
C4—C1—P1	109.9 (3)	H10B—C10—H10C	109.5
C4—C1—C2	105.5 (4)	C9—C11—H11A	109.5
C4—C1—C3	108.2 (4)	C9—C11—H11B	109.5
C1—C2—H2A	109.5	C9—C11—H11C	109.5
C1—C2—H2B	109.5	H11A—C11—H11B	109.5
C1—C2—H2C	109.5	H11A—C11—H11C	109.5
H2A—C2—H2B	109.5	H11B—C11—H11C	109.5
H2A—C2—H2C	109.5	C9—C12—H12A	109.5
H2B—C2—H2C	109.5	C9—C12—H12B	109.5
C1—C3—H3A	109.5	C9—C12—H12C	109.5
C1—C3—H3B	109.5	H12A—C12—H12B	109.5
C1—C3—H3C	109.5	H12A—C12—H12C	109.5
H3A—C3—H3B	109.5	H12B—C12—H12C	109.5
H3A—C3—H3C	109.5	O1—C13—H13A	110.7
H3B—C3—H3C	109.5	O1—C13—H13B	110.7
C1—C4—H4A	109.5	O1—C13—C14	105.4 (4)
C1—C4—H4B	109.5	H13A—C13—H13B	108.8
C1—C4—H4C	109.5	C14—C13—H13A	110.7
H4A—C4—H4B	109.5	C14—C13—H13B	110.7
H4A—C4—H4C	109.5	C13—C14—H14A	111.2
H4B—C4—H4C	109.5	C13—C14—H14B	111.2
C6—C5—P1	109.3 (3)	C13—C14—C15	102.8 (4)
C6—C5—C7	108.4 (4)	H14A—C14—H14B	109.1
C7—C5—P1	115.2 (3)	C15—C14—H14A	111.2
C8—C5—P1	107.8 (3)	C15—C14—H14B	111.2
C8—C5—C6	106.0 (4)	C14—C15—H15A	111.3
C8—C5—C7	109.8 (4)	C14—C15—H15B	111.3
C5—C6—H6A	109.5	H15A—C15—H15B	109.2
C5—C6—H6B	109.5	C16—C15—C14	102.5 (4)
C5—C6—H6C	109.5	C16—C15—H15A	111.3
H6A—C6—H6B	109.5	C16—C15—H15B	111.3
H6A—C6—H6C	109.5	O1—C16—C15	104.5 (4)
H6B—C6—H6C	109.5	O1—C16—H16A	110.9
C5—C7—H7A	109.5	O1—C16—H16B	110.9
C5—C7—H7B	109.5	C15—C16—H16A	110.9
C5—C7—H7C	109.5	C15—C16—H16B	110.9
H7A—C7—H7B	109.5	H16A—C16—H16B	108.9
H7A—C7—H7C	109.5		
			
Zn1—P1—C1—C2	−42.1 (3)	C5—P1—C1—C3	−46.3 (4)
Zn1—P1—C1—C3	−164.7 (3)	C5—P1—C1—C4	−168.4 (3)
Zn1—P1—C1—C4	73.2 (3)	C9—P1—C1—C2	−162.4 (3)
Zn1—P1—C5—C6	75.2 (4)	C9—P1—C1—C3	74.9 (4)
Zn1—P1—C5—C7	−162.5 (3)	C9—P1—C1—C4	−47.1 (4)
Zn1—P1—C5—C8	−39.6 (4)	C9—P1—C5—C6	−166.2 (3)
Zn1—O1—C13—C14	−154.5 (3)	C9—P1—C5—C7	−43.9 (4)
Zn1—O1—C16—C15	179.1 (3)	C9—P1—C5—C8	79.1 (4)
O1—C13—C14—C15	−27.6 (5)	C13—O1—C16—C15	18.7 (5)
C1—P1—C5—C6	−44.6 (4)	C13—C14—C15—C16	38.5 (5)
C1—P1—C5—C7	77.7 (4)	C14—C15—C16—O1	−35.4 (5)
C1—P1—C5—C8	−159.3 (3)	C16—O1—C13—C14	5.7 (5)
C5—P1—C1—C2	76.3 (3)		

### (2) Dichlorido(tetrahydrofuran-κO)(tri-tert-butylphosphane-κP)zinc Hydrogen-bond geometry (Å, º)

**Table:**

*D*—H···*A*	*D*—H	H···*A*	*D*···*A*	*D*—H···*A*
C12—H12*A*···Cl2^i^	0.98	2.90	3.819 (5)	157
C15—H15*A*···Cl1^ii^	0.99	2.94	3.747 (5)	140

Symmetry codes: (i) −*x*+3/2, *y*+1/2, *z*−1/2; (ii) −*x*+2, −*y*, *z*−1/2.

### (3) Tri-tert-butylphosphonium aquatrichloridozincate 1,2-dichloroethane monosolvate Crystal data

**Table:**

(C_12_H_28_P)[ZnCl_3_(H_2_O)]·C_2_H_4_Cl_2_	*F*(000) = 3072
*M**_r_* = 492.00	*D*_x_ = 1.384 Mg m^−^^3^
Monoclinic, *P*2_1_/*n*	Cu *K*α radiation, λ = 1.54187 Å
*a* = 25.3722 (5) Å	Cell parameters from 2316 reflections
*b* = 8.5841 (2) Å	θ = 23.1–67.3°
*c* = 32.912 (2) Å	µ = 7.28 mm^−^^1^
β = 98.909 (7)°	*T* = 123 K
*V* = 7081.7 (6) Å^3^	Needle, colourless
*Z* = 12	0.18 × 0.05 × 0.02 mm

### (3) Tri-tert-butylphosphonium aquatrichloridozincate 1,2-dichloroethane monosolvate Data collection

**Table:**

Rigaku CCD area-detector diffractometer	11604 independent reflections
Radiation source: sealed tube	7131 reflections with *I* > 2σ(*I*)
Focusing graphite monochromator	*R*_int_ = 0.083
Detector resolution: 22.2222 pixels mm^-1^	θ_max_ = 63.7°, θ_min_ = 6.5°
ω and φ scans	*h* = −29→29
Absorption correction: multi-scan (CrystalClear; Rigaku, 2010)	*k* = −9→9
*T*_min_ = 0.354, *T*_max_ = 0.868	*l* = −37→38
67698 measured reflections	

### (3) Tri-tert-butylphosphonium aquatrichloridozincate 1,2-dichloroethane monosolvate Refinement

**Table:**

Refinement on *F*^2^	462 restraints
Least-squares matrix: full	Hydrogen site location: mixed
*R*[*F*^2^ > 2σ(*F*^2^)] = 0.049	H atoms treated by a mixture of independent and constrained refinement
*wR*(*F*^2^) = 0.142	*w* = 1/[σ^2^(*F*_o_^2^) + (0.0718*P*)^2^] where *P* = (*F*_o_^2^ + 2*F*_c_^2^)/3
*S* = 0.98	(Δ/σ)_max_ = 0.002
11604 reflections	Δρ_max_ = 0.51 e Å^−^^3^
760 parameters	Δρ_min_ = −0.70 e Å^−^^3^

### (3) Tri-tert-butylphosphonium aquatrichloridozincate 1,2-dichloroethane monosolvate Special details

**Table:**

Geometry. All e.s.d.'s (except the e.s.d. in the dihedral angle between two l.s. planes) are estimated using the full covariance matrix. The cell e.s.d.'s are taken into account individually in the estimation of e.s.d.'s in distances, angles and torsion angles; correlations between e.s.d.'s in cell parameters are only used when they are defined by crystal symmetry. An approximate (isotropic) treatment of cell e.s.d.'s is used for estimating e.s.d.'s involving l.s. planes.

### (3) Tri-tert-butylphosphonium aquatrichloridozincate 1,2-dichloroethane monosolvate Fractional atomic coordinates and isotropic or equivalent isotropic displacement parameters (Å^2^)

**Table:**

	*x*	*y*	*z*	*U*_iso_*/*U*_eq_	Occ. (<1)
Zn1	0.29184 (2)	−0.04314 (6)	0.78814 (2)	0.03885 (16)	
Cl1	0.25307 (4)	0.14800 (11)	0.82087 (3)	0.0522 (3)	
Cl2	0.28227 (4)	−0.27034 (11)	0.82132 (3)	0.0457 (3)	
Cl3	0.37414 (4)	0.00389 (13)	0.77610 (3)	0.0548 (3)	
O1	0.24204 (11)	−0.0627 (3)	0.73407 (8)	0.0445 (7)	
H1OA	0.2483 (16)	0.017 (3)	0.7219 (11)	0.053*	
H1OB	0.2458 (16)	−0.137 (3)	0.7191 (10)	0.053*	
Zn2	0.71198 (2)	−0.04606 (6)	0.87883 (2)	0.03871 (16)	
Cl4	0.63093 (4)	0.01180 (13)	0.89180 (3)	0.0547 (3)	
Cl5	0.71685 (4)	−0.27780 (11)	0.84662 (3)	0.0485 (3)	
Cl6	0.75106 (4)	0.13977 (11)	0.84448 (3)	0.0516 (3)	
O2	0.76256 (11)	−0.0670 (3)	0.93254 (8)	0.0437 (7)	
H2OA	0.7606 (16)	0.016 (3)	0.9446 (11)	0.052*	
H2OB	0.7594 (15)	−0.146 (3)	0.9460 (10)	0.052*	
Zn3	0.71002 (2)	−0.04753 (6)	0.54563 (2)	0.03781 (16)	
Cl7	0.62855 (4)	0.00691 (12)	0.55823 (3)	0.0513 (3)	
Cl8	0.75010 (4)	0.14341 (11)	0.51378 (3)	0.0474 (3)	
Cl9	0.71658 (4)	−0.27393 (11)	0.51121 (3)	0.0453 (3)	
O3	0.75974 (11)	−0.0739 (3)	0.59969 (8)	0.0426 (7)	
H3OA	0.7567 (16)	0.010 (3)	0.6128 (11)	0.051*	
H3OB	0.7559 (15)	−0.155 (3)	0.6128 (10)	0.051*	
Cl1B	0.0853 (6)	−0.0049 (19)	0.8119 (4)	0.059 (2)	0.52 (3)
Cl2B	0.0469 (4)	0.0155 (16)	0.7101 (4)	0.0494 (17)	0.52 (3)
C37B	0.1183 (7)	−0.091 (2)	0.7729 (4)	0.057 (3)	0.52 (3)
H37A	0.1563	−0.1081	0.7842	0.068*	0.52 (3)
H37B	0.1019	−0.1930	0.7649	0.068*	0.52 (3)
C38B	0.1147 (5)	0.011 (2)	0.7355 (4)	0.054 (3)	0.52 (3)
H38A	0.1380	−0.0311	0.7166	0.065*	0.52 (3)
H38B	0.1268	0.1174	0.7436	0.065*	0.52 (3)
Cl1A	0.0777 (6)	−0.033 (2)	0.8115 (4)	0.057 (2)	0.48 (3)
Cl2A	0.0519 (5)	0.058 (2)	0.7128 (5)	0.058 (2)	0.48 (3)
C37A	0.1256 (7)	−0.057 (2)	0.7772 (5)	0.058 (3)	0.48 (3)
H37C	0.1622	−0.0472	0.7925	0.070*	0.48 (3)
H37D	0.1220	−0.1624	0.7645	0.070*	0.48 (3)
C38A	0.1159 (5)	0.065 (2)	0.7445 (5)	0.056 (3)	0.48 (3)
H38C	0.1437	0.0548	0.7265	0.067*	0.48 (3)
H38D	0.1203	0.1682	0.7578	0.067*	0.48 (3)
Cl3B	0.95522 (14)	0.0663 (5)	0.95863 (12)	0.0614 (8)	0.881 (7)
Cl4B	0.9273 (2)	−0.0185 (7)	0.85820 (13)	0.0625 (11)	0.881 (7)
C39B	0.89081 (19)	0.0740 (7)	0.92710 (18)	0.0607 (16)	0.881 (7)
H39A	0.8629	0.0644	0.9449	0.073*	0.881 (7)
H39B	0.8864	0.1767	0.9133	0.073*	0.881 (7)
C40B	0.88298 (18)	−0.0527 (7)	0.89497 (14)	0.0564 (15)	0.881 (7)
H40A	0.8456	−0.0526	0.8809	0.068*	0.881 (7)
H40B	0.8906	−0.1556	0.9082	0.068*	0.881 (7)
Cl4A	0.9330 (16)	−0.044 (5)	0.8654 (10)	0.051 (4)	0.119 (7)
Cl3A	0.9579 (9)	0.017 (3)	0.9553 (7)	0.035 (4)	0.119 (7)
C39A	0.8883 (10)	0.007 (6)	0.9349 (10)	0.055 (3)	0.119 (7)
H39C	0.8746	−0.1005	0.9353	0.066*	0.119 (7)
H39D	0.8669	0.0762	0.9502	0.066*	0.119 (7)
C40A	0.8882 (14)	0.064 (5)	0.8920 (9)	0.057 (3)	0.119 (7)
H40C	0.8985	0.1751	0.8928	0.068*	0.119 (7)
H40D	0.8516	0.0555	0.8766	0.068*	0.119 (7)
Cl5A	0.9498 (6)	0.027 (2)	0.6225 (4)	0.052 (2)	0.38 (3)
Cl6A	0.9263 (6)	−0.038 (2)	0.5236 (4)	0.060 (3)	0.38 (3)
C41A	0.8869 (6)	0.053 (3)	0.5893 (6)	0.057 (3)	0.38 (3)
H41A	0.8578	0.0477	0.6061	0.069*	0.38 (3)
H41B	0.8862	0.1578	0.5768	0.069*	0.38 (3)
C42A	0.8763 (10)	−0.064 (3)	0.5558 (6)	0.060 (3)	0.38 (3)
H42A	0.8403	−0.0486	0.5398	0.072*	0.38 (3)
H42B	0.8782	−0.1711	0.5673	0.072*	0.38 (3)
Cl5B	0.9580 (3)	−0.0244 (15)	0.6235 (3)	0.0596 (16)	0.62 (3)
Cl6B	0.9182 (4)	−0.0052 (12)	0.5223 (3)	0.0643 (18)	0.62 (3)
C41B	0.8897 (3)	−0.0012 (19)	0.5993 (4)	0.058 (2)	0.62 (3)
H41C	0.8654	−0.0432	0.6174	0.069*	0.62 (3)
H41D	0.8815	0.1108	0.5946	0.069*	0.62 (3)
C42B	0.8817 (6)	−0.0858 (19)	0.5595 (4)	0.062 (3)	0.62 (3)
H42C	0.8432	−0.0850	0.5481	0.075*	0.62 (3)
H42D	0.8926	−0.1957	0.5646	0.075*	0.62 (3)
P1	0.38352 (4)	0.01636 (11)	0.90951 (3)	0.0318 (2)	
H1P	0.3525 (12)	−0.005 (3)	0.8743 (8)	0.038*	
C1	0.39955 (14)	0.2288 (4)	0.91048 (11)	0.0388 (9)	
C2	0.44827 (15)	0.2657 (5)	0.94268 (12)	0.0529 (11)	
H2A	0.4796	0.2118	0.9355	0.063*	
H2B	0.4417	0.2308	0.9698	0.063*	
H2C	0.4547	0.3784	0.9433	0.063*	
C3	0.35169 (14)	0.3250 (4)	0.92003 (12)	0.0477 (10)	
H3A	0.3449	0.3006	0.9479	0.057*	
H3B	0.3200	0.2994	0.9001	0.057*	
H3C	0.3597	0.4363	0.9182	0.057*	
C4	0.40893 (16)	0.2786 (5)	0.86721 (11)	0.0533 (11)	
H4A	0.4111	0.3924	0.8660	0.064*	
H4B	0.3793	0.2419	0.8468	0.064*	
H4C	0.4424	0.2331	0.8614	0.064*	
C5	0.34024 (14)	−0.0466 (4)	0.94810 (10)	0.0366 (9)	
C6	0.36314 (16)	0.0051 (5)	0.99169 (12)	0.0508 (11)	
H6A	0.3400	−0.0318	1.0109	0.061*	
H6B	0.3652	0.1191	0.9927	0.061*	
H6C	0.3990	−0.0388	0.9994	0.061*	
C7	0.33375 (14)	−0.2249 (4)	0.94671 (11)	0.0464 (10)	
H7A	0.3685	−0.2742	0.9556	0.056*	
H7B	0.3198	−0.2575	0.9186	0.056*	
H7C	0.3088	−0.2565	0.9651	0.056*	
C8	0.28426 (14)	0.0216 (4)	0.93549 (12)	0.0439 (10)	
H8A	0.2597	−0.0254	0.9522	0.053*	
H8B	0.2719	−0.0008	0.9064	0.053*	
H8C	0.2854	0.1346	0.9398	0.053*	
C9	0.44243 (13)	−0.1135 (4)	0.90654 (11)	0.0362 (9)	
C10	0.47453 (15)	−0.1455 (5)	0.94889 (11)	0.0546 (12)	
H10A	0.5052	−0.2119	0.9459	0.065*	
H10B	0.4518	−0.1985	0.9661	0.065*	
H10C	0.4872	−0.0468	0.9618	0.065*	
C11	0.42058 (15)	−0.2665 (4)	0.88554 (12)	0.0520 (11)	
H11A	0.4504	−0.3327	0.8806	0.062*	
H11B	0.3982	−0.2424	0.8593	0.062*	
H11C	0.3993	−0.3215	0.9034	0.062*	
C12	0.47862 (14)	−0.0434 (5)	0.87794 (11)	0.0480 (11)	
H12A	0.4948	0.0528	0.8901	0.058*	
H12B	0.4574	−0.0202	0.8511	0.058*	
H12C	0.5067	−0.1181	0.8743	0.058*	
P2	0.61653 (4)	0.00937 (11)	0.75750 (3)	0.0357 (3)	
H2P	0.6500 (12)	−0.002 (4)	0.7919 (9)	0.043*	
C13	0.56962 (15)	0.1673 (5)	0.76841 (12)	0.0480 (11)	
C14	0.52888 (15)	0.1050 (5)	0.79467 (12)	0.0599 (12)	
H14A	0.5067	0.1911	0.8019	0.072*	
H14B	0.5479	0.0576	0.8198	0.072*	
H14C	0.5061	0.0266	0.7790	0.072*	
C15	0.53920 (16)	0.2341 (5)	0.72847 (12)	0.0589 (12)	
H15A	0.5169	0.1526	0.7137	0.071*	
H15B	0.5647	0.2723	0.7112	0.071*	
H15C	0.5165	0.3204	0.7349	0.071*	
C16	0.60288 (16)	0.2926 (4)	0.79426 (12)	0.0555 (12)	
H16A	0.5790	0.3716	0.8029	0.067*	
H16B	0.6272	0.3418	0.7777	0.067*	
H16C	0.6235	0.2440	0.8186	0.067*	
C17	0.58579 (17)	−0.1869 (5)	0.74906 (12)	0.0550 (11)	
C18	0.57213 (17)	−0.2461 (5)	0.79071 (12)	0.0656 (13)	
H18A	0.5615	−0.3559	0.7881	0.079*	
H18B	0.5427	−0.1844	0.7984	0.079*	
H18C	0.6035	−0.2355	0.8120	0.079*	
C19	0.53501 (17)	−0.1839 (5)	0.71654 (13)	0.0711 (14)	
H19A	0.5440	−0.1466	0.6903	0.085*	
H19B	0.5087	−0.1139	0.7257	0.085*	
H19C	0.5201	−0.2892	0.7130	0.085*	
C20	0.62687 (18)	−0.3035 (4)	0.73614 (12)	0.0641 (13)	
H20A	0.6590	−0.3043	0.7570	0.077*	
H20B	0.6364	−0.2717	0.7096	0.077*	
H20C	0.6112	−0.4081	0.7337	0.077*	
C21	0.66109 (15)	0.0665 (4)	0.71933 (11)	0.0443 (10)	
C22	0.71302 (15)	−0.0295 (5)	0.72902 (12)	0.0494 (11)	
H22A	0.7049	−0.1403	0.7243	0.059*	
H22B	0.7289	−0.0135	0.7578	0.059*	
H22C	0.7383	0.0042	0.7111	0.059*	
C23	0.67727 (15)	0.2390 (4)	0.72459 (11)	0.0487 (11)	
H23A	0.7020	0.2650	0.7055	0.058*	
H23B	0.6947	0.2571	0.7529	0.058*	
H23C	0.6454	0.3046	0.7187	0.058*	
C24	0.63430 (17)	0.0377 (5)	0.67480 (12)	0.0558 (12)	
H24A	0.6005	0.0948	0.6695	0.067*	
H24B	0.6275	−0.0740	0.6706	0.067*	
H24C	0.6579	0.0738	0.6558	0.067*	
P3	0.61839 (4)	0.01954 (11)	0.42420 (3)	0.0311 (2)	
H3P	0.6475 (12)	−0.009 (3)	0.4597 (8)	0.037*	
C25	0.55825 (14)	−0.1067 (4)	0.42541 (11)	0.0376 (9)	
C26	0.57715 (15)	−0.2653 (4)	0.44388 (12)	0.0525 (11)	
H26A	0.5990	−0.2495	0.4708	0.063*	
H26B	0.5984	−0.3181	0.4255	0.063*	
H26C	0.5461	−0.3295	0.4470	0.063*	
C27	0.52264 (14)	−0.0382 (5)	0.45445 (11)	0.0474 (11)	
H27A	0.4927	−0.1089	0.4561	0.057*	
H27B	0.5089	0.0632	0.4440	0.057*	
H27C	0.5435	−0.0250	0.4819	0.057*	
C28	0.52641 (15)	−0.1280 (5)	0.38235 (11)	0.0515 (11)	
H28A	0.4946	−0.1907	0.3841	0.062*	
H28B	0.5486	−0.1811	0.3648	0.062*	
H28C	0.5157	−0.0258	0.3706	0.062*	
C29	0.60386 (14)	0.2337 (4)	0.42467 (11)	0.0370 (9)	
C30	0.65241 (14)	0.3281 (4)	0.41584 (12)	0.0459 (10)	
H30A	0.6589	0.3063	0.3878	0.055*	
H30B	0.6839	0.2985	0.4355	0.055*	
H30C	0.6454	0.4395	0.4186	0.055*	
C31	0.55516 (15)	0.2753 (4)	0.39264 (12)	0.0497 (11)	
H31A	0.5235	0.2229	0.3997	0.060*	
H31B	0.5614	0.2412	0.3654	0.060*	
H31C	0.5496	0.3883	0.3925	0.060*	
C32	0.59471 (15)	0.2798 (4)	0.46828 (11)	0.0493 (11)	
H32A	0.5905	0.3930	0.4697	0.059*	
H32B	0.6254	0.2471	0.4883	0.059*	
H32C	0.5624	0.2285	0.4746	0.059*	
C33	0.66206 (14)	−0.0426 (4)	0.38595 (11)	0.0371 (9)	
C34	0.66708 (15)	−0.2203 (4)	0.38602 (11)	0.0465 (10)	
H34A	0.6323	−0.2666	0.3756	0.056*	
H34B	0.6792	−0.2567	0.4141	0.056*	
H34C	0.6930	−0.2513	0.3683	0.056*	
C35	0.71849 (14)	0.0222 (4)	0.39901 (12)	0.0415 (10)	
H35A	0.7300	0.0018	0.4283	0.050*	
H35B	0.7184	0.1348	0.3940	0.050*	
H35C	0.7431	−0.0285	0.3830	0.050*	
C36	0.63950 (16)	0.0148 (5)	0.34197 (11)	0.0517 (11)	
H36A	0.6623	−0.0226	0.3226	0.062*	
H36B	0.6386	0.1289	0.3416	0.062*	
H36C	0.6033	−0.0257	0.3340	0.062*	

### (3) Tri-tert-butylphosphonium aquatrichloridozincate 1,2-dichloroethane monosolvate Atomic displacement parameters (Å^2^)

**Table:**

	*U*^11^	*U*^22^	*U*^33^	*U*^12^	*U*^13^	*U*^23^
Zn1	0.0415 (3)	0.0430 (3)	0.0322 (3)	−0.0021 (2)	0.0062 (2)	0.0010 (2)
Cl1	0.0715 (7)	0.0485 (6)	0.0376 (6)	0.0113 (5)	0.0117 (5)	−0.0014 (4)
Cl2	0.0556 (6)	0.0456 (6)	0.0373 (5)	−0.0081 (5)	0.0119 (5)	0.0038 (4)
Cl3	0.0462 (6)	0.0697 (7)	0.0492 (7)	−0.0127 (5)	0.0097 (5)	0.0043 (5)
O1	0.0489 (17)	0.048 (2)	0.0355 (17)	0.0021 (15)	0.0033 (14)	−0.0030 (13)
Zn2	0.0400 (3)	0.0443 (3)	0.0320 (3)	0.0022 (2)	0.0059 (2)	0.0003 (2)
Cl4	0.0430 (6)	0.0744 (8)	0.0478 (7)	0.0118 (5)	0.0102 (5)	−0.0053 (5)
Cl5	0.0595 (7)	0.0472 (6)	0.0399 (6)	0.0085 (5)	0.0115 (5)	−0.0028 (4)
Cl6	0.0661 (7)	0.0491 (6)	0.0405 (6)	−0.0072 (5)	0.0112 (5)	0.0030 (5)
O2	0.0476 (16)	0.0423 (19)	0.0396 (18)	−0.0021 (15)	0.0012 (13)	0.0041 (13)
Zn3	0.0372 (3)	0.0435 (3)	0.0331 (3)	0.0026 (2)	0.0067 (2)	−0.0004 (2)
Cl7	0.0412 (6)	0.0630 (7)	0.0513 (7)	0.0091 (5)	0.0119 (5)	0.0004 (5)
Cl8	0.0590 (7)	0.0461 (6)	0.0384 (5)	−0.0053 (5)	0.0120 (5)	0.0014 (4)
Cl9	0.0551 (6)	0.0455 (6)	0.0376 (5)	0.0071 (5)	0.0142 (5)	−0.0028 (4)
O3	0.0454 (16)	0.0445 (19)	0.0369 (17)	−0.0014 (15)	0.0029 (13)	0.0024 (12)
Cl1B	0.065 (4)	0.068 (4)	0.043 (3)	0.003 (3)	0.009 (2)	−0.011 (3)
Cl2B	0.039 (2)	0.067 (5)	0.043 (2)	−0.005 (2)	0.0073 (17)	0.000 (3)
C37B	0.045 (4)	0.073 (6)	0.051 (4)	0.006 (4)	0.004 (4)	−0.008 (4)
C38B	0.040 (4)	0.078 (7)	0.045 (4)	−0.002 (4)	0.006 (3)	−0.009 (4)
Cl1A	0.056 (3)	0.073 (5)	0.041 (3)	0.004 (3)	0.006 (2)	0.014 (3)
Cl2A	0.049 (3)	0.080 (6)	0.044 (3)	−0.009 (3)	0.003 (2)	0.012 (4)
C37A	0.046 (4)	0.075 (6)	0.052 (4)	0.003 (4)	0.004 (4)	−0.004 (4)
C38A	0.043 (4)	0.072 (6)	0.052 (5)	−0.002 (4)	0.010 (4)	0.001 (4)
Cl3B	0.0523 (11)	0.089 (2)	0.0457 (12)	−0.0071 (14)	0.0169 (9)	−0.0158 (14)
Cl4B	0.067 (2)	0.085 (2)	0.0352 (16)	0.0038 (14)	0.0082 (15)	−0.0016 (12)
C39B	0.043 (3)	0.083 (4)	0.059 (3)	−0.005 (3)	0.015 (2)	−0.014 (3)
C40B	0.045 (3)	0.070 (4)	0.054 (3)	−0.005 (3)	0.009 (2)	−0.007 (2)
Cl4A	0.051 (7)	0.061 (9)	0.040 (9)	0.017 (6)	0.000 (7)	−0.007 (7)
Cl3A	0.022 (5)	0.060 (9)	0.021 (5)	0.015 (5)	−0.004 (4)	−0.027 (5)
C39A	0.037 (5)	0.076 (6)	0.051 (5)	0.002 (5)	0.003 (5)	−0.009 (5)
C40A	0.044 (5)	0.074 (6)	0.051 (5)	0.003 (5)	0.003 (5)	−0.011 (5)
Cl5A	0.044 (4)	0.078 (6)	0.034 (2)	−0.016 (3)	0.009 (3)	−0.003 (3)
Cl6A	0.049 (3)	0.093 (6)	0.038 (4)	−0.005 (4)	0.010 (3)	−0.025 (4)
C41A	0.040 (4)	0.085 (7)	0.049 (5)	−0.001 (5)	0.016 (4)	−0.007 (5)
C42A	0.042 (5)	0.087 (6)	0.049 (5)	0.001 (4)	0.004 (4)	0.003 (4)
Cl5B	0.0352 (17)	0.096 (5)	0.0475 (15)	0.000 (2)	0.0064 (12)	0.006 (3)
Cl6B	0.069 (4)	0.079 (3)	0.045 (2)	−0.012 (3)	0.0077 (18)	0.016 (2)
C41B	0.040 (3)	0.091 (6)	0.042 (4)	0.003 (4)	0.006 (3)	0.005 (4)
C42B	0.044 (4)	0.090 (5)	0.052 (4)	−0.007 (4)	0.002 (3)	0.011 (3)
P1	0.0305 (6)	0.0356 (6)	0.0302 (6)	−0.0023 (4)	0.0080 (5)	−0.0020 (4)
C1	0.037 (2)	0.037 (2)	0.043 (2)	−0.0040 (18)	0.0054 (18)	−0.0059 (18)
C2	0.050 (3)	0.048 (3)	0.060 (3)	−0.005 (2)	0.008 (2)	−0.008 (2)
C3	0.044 (2)	0.038 (2)	0.062 (3)	0.0019 (19)	0.009 (2)	−0.001 (2)
C4	0.062 (3)	0.046 (3)	0.055 (3)	−0.005 (2)	0.018 (2)	0.006 (2)
C5	0.042 (2)	0.041 (2)	0.030 (2)	−0.0003 (19)	0.0141 (18)	0.0025 (17)
C6	0.049 (3)	0.066 (3)	0.040 (3)	0.005 (2)	0.013 (2)	0.002 (2)
C7	0.042 (2)	0.047 (3)	0.053 (3)	0.000 (2)	0.017 (2)	0.0093 (19)
C8	0.034 (2)	0.055 (3)	0.045 (3)	0.0057 (19)	0.014 (2)	0.0081 (19)
C9	0.030 (2)	0.040 (2)	0.041 (2)	0.0006 (17)	0.0104 (18)	−0.0001 (18)
C10	0.046 (3)	0.067 (3)	0.051 (3)	0.017 (2)	0.012 (2)	0.007 (2)
C11	0.047 (3)	0.051 (3)	0.062 (3)	0.002 (2)	0.020 (2)	−0.007 (2)
C12	0.037 (2)	0.061 (3)	0.050 (3)	0.002 (2)	0.018 (2)	0.001 (2)
P2	0.0354 (6)	0.0385 (6)	0.0332 (6)	−0.0001 (4)	0.0051 (5)	0.0025 (4)
C13	0.043 (2)	0.056 (3)	0.047 (3)	0.009 (2)	0.014 (2)	0.013 (2)
C14	0.048 (3)	0.075 (3)	0.061 (3)	0.014 (2)	0.024 (2)	0.021 (2)
C15	0.053 (3)	0.070 (3)	0.056 (3)	0.016 (2)	0.014 (2)	0.016 (2)
C16	0.070 (3)	0.049 (3)	0.051 (3)	0.015 (2)	0.023 (2)	0.002 (2)
C17	0.062 (3)	0.047 (3)	0.051 (3)	−0.012 (2)	−0.007 (2)	0.007 (2)
C18	0.068 (3)	0.062 (3)	0.063 (3)	−0.021 (3)	0.000 (3)	0.012 (2)
C19	0.071 (3)	0.071 (3)	0.064 (3)	−0.025 (3)	−0.013 (3)	0.004 (3)
C20	0.090 (4)	0.039 (3)	0.059 (3)	−0.002 (2)	−0.003 (3)	−0.004 (2)
C21	0.050 (3)	0.048 (3)	0.037 (2)	0.003 (2)	0.013 (2)	0.0036 (18)
C22	0.044 (3)	0.061 (3)	0.045 (3)	0.013 (2)	0.014 (2)	0.000 (2)
C23	0.048 (3)	0.052 (3)	0.050 (3)	−0.004 (2)	0.019 (2)	0.003 (2)
C24	0.061 (3)	0.067 (3)	0.038 (3)	0.003 (2)	0.003 (2)	0.004 (2)
P3	0.0293 (5)	0.0349 (5)	0.0297 (6)	0.0011 (4)	0.0070 (4)	0.0016 (4)
C25	0.035 (2)	0.040 (2)	0.040 (2)	−0.0016 (18)	0.0122 (18)	0.0009 (18)
C26	0.043 (3)	0.052 (3)	0.066 (3)	−0.005 (2)	0.022 (2)	0.008 (2)
C27	0.036 (2)	0.061 (3)	0.048 (3)	0.000 (2)	0.015 (2)	−0.002 (2)
C28	0.042 (2)	0.064 (3)	0.049 (3)	−0.013 (2)	0.005 (2)	−0.008 (2)
C29	0.034 (2)	0.034 (2)	0.042 (2)	0.0028 (17)	0.0040 (18)	0.0025 (17)
C30	0.044 (2)	0.033 (2)	0.061 (3)	0.0018 (19)	0.010 (2)	0.0046 (19)
C31	0.044 (3)	0.048 (3)	0.056 (3)	0.003 (2)	0.004 (2)	0.006 (2)
C32	0.046 (3)	0.046 (3)	0.058 (3)	0.005 (2)	0.013 (2)	−0.004 (2)
C33	0.035 (2)	0.043 (2)	0.035 (2)	−0.0020 (19)	0.0113 (18)	−0.0051 (18)
C34	0.046 (2)	0.044 (3)	0.053 (3)	−0.001 (2)	0.018 (2)	−0.0106 (19)
C35	0.036 (2)	0.045 (2)	0.046 (3)	−0.0022 (19)	0.0138 (19)	−0.0045 (19)
C36	0.054 (3)	0.069 (3)	0.033 (2)	−0.006 (2)	0.011 (2)	0.001 (2)

### (3) Tri-tert-butylphosphonium aquatrichloridozincate 1,2-dichloroethane monosolvate Geometric parameters (Å, º)

**Table:**

Zn1—Cl1	2.2690 (10)	C9—C12	1.536 (5)
Zn1—Cl2	2.2666 (10)	C10—H10A	0.9800
Zn1—Cl3	2.2219 (11)	C10—H10B	0.9800
Zn1—O1	2.024 (3)	C10—H10C	0.9800
O1—H1OA	0.817 (18)	C11—H11A	0.9800
O1—H1OB	0.821 (18)	C11—H11B	0.9800
Zn2—Cl4	2.2203 (11)	C11—H11C	0.9800
Zn2—Cl5	2.2666 (10)	C12—H12A	0.9800
Zn2—Cl6	2.2699 (10)	C12—H12B	0.9800
Zn2—O2	2.025 (3)	C12—H12C	0.9800
O2—H2OA	0.822 (18)	P2—H2P	1.31 (3)
O2—H2OB	0.821 (18)	P2—C13	1.875 (4)
Zn3—Cl7	2.2199 (10)	P2—C17	1.860 (4)
Zn3—Cl8	2.2695 (10)	P2—C21	1.881 (4)
Zn3—Cl9	2.2686 (10)	C13—C14	1.542 (5)
Zn3—O3	2.028 (3)	C13—C15	1.529 (5)
O3—H3OA	0.847 (18)	C13—C16	1.539 (5)
O3—H3OB	0.833 (18)	C14—H14A	0.9800
Cl1B—C37B	1.797 (7)	C14—H14B	0.9800
Cl2B—C38B	1.793 (6)	C14—H14C	0.9800
C37B—H37A	0.9900	C15—H15A	0.9800
C37B—H37B	0.9900	C15—H15B	0.9800
C37B—C38B	1.496 (7)	C15—H15C	0.9800
C38B—H38A	0.9900	C16—H16A	0.9800
C38B—H38B	0.9900	C16—H16B	0.9800
Cl1A—C37A	1.798 (7)	C16—H16C	0.9800
Cl2A—C38A	1.789 (7)	C17—C18	1.551 (5)
C37A—H37C	0.9900	C17—C19	1.542 (5)
C37A—H37D	0.9900	C17—C20	1.551 (5)
C37A—C38A	1.495 (8)	C18—H18A	0.9800
C38A—H38C	0.9900	C18—H18B	0.9800
C38A—H38D	0.9900	C18—H18C	0.9800
Cl3B—C39B	1.796 (4)	C19—H19A	0.9800
Cl4B—C40B	1.799 (5)	C19—H19B	0.9800
C39B—H39A	0.9900	C19—H19C	0.9800
C39B—H39B	0.9900	C20—H20A	0.9800
C39B—C40B	1.509 (6)	C20—H20B	0.9800
C40B—H40A	0.9900	C20—H20C	0.9800
C40B—H40B	0.9900	C21—C22	1.545 (5)
Cl4A—C40A	1.797 (8)	C21—C23	1.538 (5)
Cl3A—C39A	1.792 (8)	C21—C24	1.537 (5)
C39A—H39C	0.9900	C22—H22A	0.9800
C39A—H39D	0.9900	C22—H22B	0.9800
C39A—C40A	1.493 (9)	C22—H22C	0.9800
C40A—H40C	0.9900	C23—H23A	0.9800
C40A—H40D	0.9900	C23—H23B	0.9800
Cl5A—C41A	1.803 (7)	C23—H23C	0.9800
Cl6A—C42A	1.790 (7)	C24—H24A	0.9800
C41A—H41A	0.9900	C24—H24B	0.9800
C41A—H41B	0.9900	C24—H24C	0.9800
C41A—C42A	1.486 (8)	P3—H3P	1.31 (3)
C42A—H42A	0.9900	P3—C25	1.877 (4)
C42A—H42B	0.9900	P3—C29	1.876 (4)
Cl5B—C41B	1.803 (6)	P3—C33	1.879 (3)
Cl6B—C42B	1.787 (6)	C25—C26	1.537 (5)
C41B—H41C	0.9900	C25—C27	1.531 (5)
C41B—H41D	0.9900	C25—C28	1.530 (5)
C41B—C42B	1.484 (7)	C26—H26A	0.9800
C42B—H42C	0.9900	C26—H26B	0.9800
C42B—H42D	0.9900	C26—H26C	0.9800
P1—H1P	1.31 (3)	C27—H27A	0.9800
P1—C1	1.868 (4)	C27—H27B	0.9800
P1—C5	1.882 (3)	C27—H27C	0.9800
P1—C9	1.879 (3)	C28—H28A	0.9800
C1—C2	1.532 (5)	C28—H28B	0.9800
C1—C3	1.541 (5)	C28—H28C	0.9800
C1—C4	1.540 (5)	C29—C30	1.539 (5)
C2—H2A	0.9800	C29—C31	1.537 (5)
C2—H2B	0.9800	C29—C32	1.541 (5)
C2—H2C	0.9800	C30—H30A	0.9800
C3—H3A	0.9800	C30—H30B	0.9800
C3—H3B	0.9800	C30—H30C	0.9800
C3—H3C	0.9800	C31—H31A	0.9800
C4—H4A	0.9800	C31—H31B	0.9800
C4—H4B	0.9800	C31—H31C	0.9800
C4—H4C	0.9800	C32—H32A	0.9800
C5—C6	1.528 (5)	C32—H32B	0.9800
C5—C7	1.539 (5)	C32—H32C	0.9800
C5—C8	1.533 (5)	C33—C34	1.530 (5)
C6—H6A	0.9800	C33—C35	1.534 (5)
C6—H6B	0.9800	C33—C36	1.552 (5)
C6—H6C	0.9800	C34—H34A	0.9800
C7—H7A	0.9800	C34—H34B	0.9800
C7—H7B	0.9800	C34—H34C	0.9800
C7—H7C	0.9800	C35—H35A	0.9800
C8—H8A	0.9800	C35—H35B	0.9800
C8—H8B	0.9800	C35—H35C	0.9800
C8—H8C	0.9800	C36—H36A	0.9800
C9—C10	1.527 (5)	C36—H36B	0.9800
C9—C11	1.547 (5)	C36—H36C	0.9800
			
Cl2—Zn1—Cl1	107.87 (4)	C9—C11—H11C	109.5
Cl3—Zn1—Cl1	116.22 (4)	H11A—C11—H11B	109.5
Cl3—Zn1—Cl2	114.58 (4)	H11A—C11—H11C	109.5
O1—Zn1—Cl1	102.71 (8)	H11B—C11—H11C	109.5
O1—Zn1—Cl2	104.68 (8)	C9—C12—H12A	109.5
O1—Zn1—Cl3	109.53 (8)	C9—C12—H12B	109.5
Zn1—O1—H1OA	103 (3)	C9—C12—H12C	109.5
Zn1—O1—H1OB	118 (3)	H12A—C12—H12B	109.5
H1OA—O1—H1OB	108 (4)	H12A—C12—H12C	109.5
Cl4—Zn2—Cl5	113.92 (4)	H12B—C12—H12C	109.5
Cl4—Zn2—Cl6	115.31 (4)	C13—P2—H2P	103.5 (14)
Cl5—Zn2—Cl6	108.91 (4)	C13—P2—C21	113.85 (17)
O2—Zn2—Cl4	109.43 (8)	C17—P2—H2P	105.3 (14)
O2—Zn2—Cl5	104.91 (8)	C17—P2—C13	114.82 (19)
O2—Zn2—Cl6	103.33 (8)	C17—P2—C21	114.73 (18)
Zn2—O2—H2OA	105 (3)	C21—P2—H2P	102.7 (14)
Zn2—O2—H2OB	116 (3)	C14—C13—P2	110.9 (3)
H2OA—O2—H2OB	116 (4)	C15—C13—P2	110.9 (3)
Cl7—Zn3—Cl8	115.54 (4)	C15—C13—C14	108.6 (3)
Cl7—Zn3—Cl9	114.59 (4)	C15—C13—C16	111.9 (3)
Cl9—Zn3—Cl8	108.37 (4)	C16—C13—P2	107.5 (3)
O3—Zn3—Cl7	109.29 (8)	C16—C13—C14	106.9 (3)
O3—Zn3—Cl8	102.93 (8)	C13—C14—H14A	109.5
O3—Zn3—Cl9	104.98 (8)	C13—C14—H14B	109.5
Zn3—O3—H3OA	105 (3)	C13—C14—H14C	109.5
Zn3—O3—H3OB	116 (3)	H14A—C14—H14B	109.5
H3OA—O3—H3OB	115 (4)	H14A—C14—H14C	109.5
Cl1B—C37B—H37A	109.2	H14B—C14—H14C	109.5
Cl1B—C37B—H37B	109.2	C13—C15—H15A	109.5
H37A—C37B—H37B	107.9	C13—C15—H15B	109.5
C38B—C37B—Cl1B	112.0 (11)	C13—C15—H15C	109.5
C38B—C37B—H37A	109.2	H15A—C15—H15B	109.5
C38B—C37B—H37B	109.2	H15A—C15—H15C	109.5
Cl2B—C38B—H38A	109.8	H15B—C15—H15C	109.5
Cl2B—C38B—H38B	109.8	C13—C16—H16A	109.5
C37B—C38B—Cl2B	109.2 (10)	C13—C16—H16B	109.5
C37B—C38B—H38A	109.8	C13—C16—H16C	109.5
C37B—C38B—H38B	109.8	H16A—C16—H16B	109.5
H38A—C38B—H38B	108.3	H16A—C16—H16C	109.5
Cl1A—C37A—H37C	110.0	H16B—C16—H16C	109.5
Cl1A—C37A—H37D	110.0	C18—C17—P2	108.1 (3)
H37C—C37A—H37D	108.4	C18—C17—C20	106.4 (3)
C38A—C37A—Cl1A	108.6 (12)	C19—C17—P2	112.0 (3)
C38A—C37A—H37C	110.0	C19—C17—C18	110.0 (3)
C38A—C37A—H37D	110.0	C19—C17—C20	110.2 (4)
Cl2A—C38A—H38C	108.4	C20—C17—P2	110.0 (3)
Cl2A—C38A—H38D	108.4	C17—C18—H18A	109.5
C37A—C38A—Cl2A	115.4 (11)	C17—C18—H18B	109.5
C37A—C38A—H38C	108.4	C17—C18—H18C	109.5
C37A—C38A—H38D	108.4	H18A—C18—H18B	109.5
H38C—C38A—H38D	107.5	H18A—C18—H18C	109.5
Cl3B—C39B—H39A	109.0	H18B—C18—H18C	109.5
Cl3B—C39B—H39B	109.0	C17—C19—H19A	109.5
H39A—C39B—H39B	107.8	C17—C19—H19B	109.5
C40B—C39B—Cl3B	112.8 (4)	C17—C19—H19C	109.5
C40B—C39B—H39A	109.0	H19A—C19—H19B	109.5
C40B—C39B—H39B	109.0	H19A—C19—H19C	109.5
Cl4B—C40B—H40A	109.9	H19B—C19—H19C	109.5
Cl4B—C40B—H40B	109.9	C17—C20—H20A	109.5
C39B—C40B—Cl4B	109.0 (5)	C17—C20—H20B	109.5
C39B—C40B—H40A	109.9	C17—C20—H20C	109.5
C39B—C40B—H40B	109.9	H20A—C20—H20B	109.5
H40A—C40B—H40B	108.3	H20A—C20—H20C	109.5
Cl3A—C39A—H39C	111.5	H20B—C20—H20C	109.5
Cl3A—C39A—H39D	111.5	C22—C21—P2	107.7 (2)
H39C—C39A—H39D	109.3	C23—C21—P2	110.7 (2)
C40A—C39A—Cl3A	101 (2)	C23—C21—C22	106.6 (3)
C40A—C39A—H39C	111.5	C24—C21—P2	111.8 (3)
C40A—C39A—H39D	111.5	C24—C21—C22	110.3 (3)
Cl4A—C40A—H40C	109.0	C24—C21—C23	109.5 (3)
Cl4A—C40A—H40D	109.0	C21—C22—H22A	109.5
C39A—C40A—Cl4A	113 (3)	C21—C22—H22B	109.5
C39A—C40A—H40C	109.0	C21—C22—H22C	109.5
C39A—C40A—H40D	109.0	H22A—C22—H22B	109.5
H40C—C40A—H40D	107.8	H22A—C22—H22C	109.5
Cl5A—C41A—H41A	108.7	H22B—C22—H22C	109.5
Cl5A—C41A—H41B	108.7	C21—C23—H23A	109.5
H41A—C41A—H41B	107.6	C21—C23—H23B	109.5
C42A—C41A—Cl5A	114.2 (14)	C21—C23—H23C	109.5
C42A—C41A—H41A	108.7	H23A—C23—H23B	109.5
C42A—C41A—H41B	108.7	H23A—C23—H23C	109.5
Cl6A—C42A—H42A	110.4	H23B—C23—H23C	109.5
Cl6A—C42A—H42B	110.4	C21—C24—H24A	109.5
C41A—C42A—Cl6A	106.7 (14)	C21—C24—H24B	109.5
C41A—C42A—H42A	110.4	C21—C24—H24C	109.5
C41A—C42A—H42B	110.4	H24A—C24—H24B	109.5
H42A—C42A—H42B	108.6	H24A—C24—H24C	109.5
Cl5B—C41B—H41C	109.9	H24B—C24—H24C	109.5
Cl5B—C41B—H41D	109.9	C25—P3—H3P	102.8 (13)
H41C—C41B—H41D	108.3	C25—P3—C33	114.59 (16)
C42B—C41B—Cl5B	109.0 (9)	C29—P3—H3P	105.2 (13)
C42B—C41B—H41C	109.9	C29—P3—C25	113.83 (16)
C42B—C41B—H41D	109.9	C29—P3—C33	114.89 (16)
Cl6B—C42B—H42C	108.8	C33—P3—H3P	103.6 (13)
Cl6B—C42B—H42D	108.8	C26—C25—P3	108.3 (2)
C41B—C42B—Cl6B	113.7 (8)	C27—C25—P3	110.6 (3)
C41B—C42B—H42C	108.8	C27—C25—C26	105.7 (3)
C41B—C42B—H42D	108.8	C28—C25—P3	111.3 (2)
H42C—C42B—H42D	107.7	C28—C25—C26	110.4 (3)
C1—P1—H1P	104.7 (13)	C28—C25—C27	110.3 (3)
C1—P1—C5	114.66 (16)	C25—C26—H26A	109.5
C1—P1—C9	114.03 (16)	C25—C26—H26B	109.5
C5—P1—H1P	102.8 (14)	C25—C26—H26C	109.5
C9—P1—H1P	104.2 (14)	H26A—C26—H26B	109.5
C9—P1—C5	114.61 (16)	H26A—C26—H26C	109.5
C2—C1—P1	111.4 (3)	H26B—C26—H26C	109.5
C2—C1—C3	109.0 (3)	C25—C27—H27A	109.5
C2—C1—C4	111.0 (3)	C25—C27—H27B	109.5
C3—C1—P1	110.5 (2)	C25—C27—H27C	109.5
C4—C1—P1	108.6 (2)	H27A—C27—H27B	109.5
C4—C1—C3	106.2 (3)	H27A—C27—H27C	109.5
C1—C2—H2A	109.5	H27B—C27—H27C	109.5
C1—C2—H2B	109.5	C25—C28—H28A	109.5
C1—C2—H2C	109.5	C25—C28—H28B	109.5
H2A—C2—H2B	109.5	C25—C28—H28C	109.5
H2A—C2—H2C	109.5	H28A—C28—H28B	109.5
H2B—C2—H2C	109.5	H28A—C28—H28C	109.5
C1—C3—H3A	109.5	H28B—C28—H28C	109.5
C1—C3—H3B	109.5	C30—C29—P3	110.5 (2)
C1—C3—H3C	109.5	C30—C29—C32	106.3 (3)
H3A—C3—H3B	109.5	C31—C29—P3	111.1 (2)
H3A—C3—H3C	109.5	C31—C29—C30	109.3 (3)
H3B—C3—H3C	109.5	C31—C29—C32	111.0 (3)
C1—C4—H4A	109.5	C32—C29—P3	108.5 (2)
C1—C4—H4B	109.5	C29—C30—H30A	109.5
C1—C4—H4C	109.5	C29—C30—H30B	109.5
H4A—C4—H4B	109.5	C29—C30—H30C	109.5
H4A—C4—H4C	109.5	H30A—C30—H30B	109.5
H4B—C4—H4C	109.5	H30A—C30—H30C	109.5
C6—C5—P1	111.9 (3)	H30B—C30—H30C	109.5
C6—C5—C7	109.9 (3)	C29—C31—H31A	109.5
C6—C5—C8	110.2 (3)	C29—C31—H31B	109.5
C7—C5—P1	109.7 (2)	C29—C31—H31C	109.5
C8—C5—P1	108.7 (2)	H31A—C31—H31B	109.5
C8—C5—C7	106.3 (3)	H31A—C31—H31C	109.5
C5—C6—H6A	109.5	H31B—C31—H31C	109.5
C5—C6—H6B	109.5	C29—C32—H32A	109.5
C5—C6—H6C	109.5	C29—C32—H32B	109.5
H6A—C6—H6B	109.5	C29—C32—H32C	109.5
H6A—C6—H6C	109.5	H32A—C32—H32B	109.5
H6B—C6—H6C	109.5	H32A—C32—H32C	109.5
C5—C7—H7A	109.5	H32B—C32—H32C	109.5
C5—C7—H7B	109.5	C34—C33—P3	109.8 (2)
C5—C7—H7C	109.5	C34—C33—C35	106.7 (3)
H7A—C7—H7B	109.5	C34—C33—C36	109.7 (3)
H7A—C7—H7C	109.5	C35—C33—P3	109.3 (2)
H7B—C7—H7C	109.5	C35—C33—C36	109.9 (3)
C5—C8—H8A	109.5	C36—C33—P3	111.4 (3)
C5—C8—H8B	109.5	C33—C34—H34A	109.5
C5—C8—H8C	109.5	C33—C34—H34B	109.5
H8A—C8—H8B	109.5	C33—C34—H34C	109.5
H8A—C8—H8C	109.5	H34A—C34—H34B	109.5
H8B—C8—H8C	109.5	H34A—C34—H34C	109.5
C10—C9—P1	112.1 (2)	H34B—C34—H34C	109.5
C10—C9—C11	111.0 (3)	C33—C35—H35A	109.5
C10—C9—C12	110.2 (3)	C33—C35—H35B	109.5
C11—C9—P1	107.1 (2)	C33—C35—H35C	109.5
C12—C9—P1	110.8 (3)	H35A—C35—H35B	109.5
C12—C9—C11	105.4 (3)	H35A—C35—H35C	109.5
C9—C10—H10A	109.5	H35B—C35—H35C	109.5
C9—C10—H10B	109.5	C33—C36—H36A	109.5
C9—C10—H10C	109.5	C33—C36—H36B	109.5
H10A—C10—H10B	109.5	C33—C36—H36C	109.5
H10A—C10—H10C	109.5	H36A—C36—H36B	109.5
H10B—C10—H10C	109.5	H36A—C36—H36C	109.5
C9—C11—H11A	109.5	H36B—C36—H36C	109.5
C9—C11—H11B	109.5		
			
Cl1B—C37B—C38B—Cl2B	−68.6 (12)	C17—P2—C13—C14	36.4 (3)
Cl1A—C37A—C38A—Cl2A	−61.4 (14)	C17—P2—C13—C15	−84.4 (3)
Cl3B—C39B—C40B—Cl4B	−67.1 (6)	C17—P2—C13—C16	152.9 (2)
Cl3A—C39A—C40A—Cl4A	54 (4)	C17—P2—C21—C22	−72.8 (3)
Cl5A—C41A—C42A—Cl6A	−64 (2)	C17—P2—C21—C23	171.0 (3)
Cl5B—C41B—C42B—Cl6B	−66.1 (12)	C17—P2—C21—C24	48.6 (3)
C1—P1—C5—C6	−53.1 (3)	C21—P2—C13—C14	171.5 (3)
C1—P1—C5—C7	−175.4 (2)	C21—P2—C13—C15	50.7 (3)
C1—P1—C5—C8	68.7 (3)	C21—P2—C13—C16	−72.0 (3)
C1—P1—C9—C10	84.8 (3)	C21—P2—C17—C18	155.5 (3)
C1—P1—C9—C11	−153.2 (2)	C21—P2—C17—C19	−83.1 (3)
C1—P1—C9—C12	−38.7 (3)	C21—P2—C17—C20	39.7 (3)
C5—P1—C1—C2	85.5 (3)	C25—P3—C29—C30	−169.9 (2)
C5—P1—C1—C3	−35.8 (3)	C25—P3—C29—C31	−48.4 (3)
C5—P1—C1—C4	−152.0 (2)	C25—P3—C29—C32	73.9 (3)
C5—P1—C9—C10	−50.2 (3)	C25—P3—C33—C34	−40.7 (3)
C5—P1—C9—C11	71.8 (3)	C25—P3—C33—C35	−157.4 (2)
C5—P1—C9—C12	−173.7 (2)	C25—P3—C33—C36	81.0 (3)
C9—P1—C1—C2	−49.5 (3)	C29—P3—C25—C26	−155.1 (2)
C9—P1—C1—C3	−170.7 (2)	C29—P3—C25—C27	−39.6 (3)
C9—P1—C1—C4	73.1 (3)	C29—P3—C25—C28	83.4 (3)
C9—P1—C5—C6	81.5 (3)	C29—P3—C33—C34	−175.3 (2)
C9—P1—C5—C7	−40.7 (3)	C29—P3—C33—C35	68.0 (3)
C9—P1—C5—C8	−156.6 (2)	C29—P3—C33—C36	−53.6 (3)
C13—P2—C17—C18	−69.8 (3)	C33—P3—C25—C26	69.8 (3)
C13—P2—C17—C19	51.6 (4)	C33—P3—C25—C27	−174.7 (2)
C13—P2—C17—C20	174.4 (3)	C33—P3—C25—C28	−51.7 (3)
C13—P2—C21—C22	152.0 (3)	C33—P3—C29—C30	−34.9 (3)
C13—P2—C21—C23	35.8 (3)	C33—P3—C29—C31	86.6 (3)
C13—P2—C21—C24	−86.5 (3)	C33—P3—C29—C32	−151.1 (2)

### (3) Tri-tert-butylphosphonium aquatrichloridozincate 1,2-dichloroethane monosolvate Hydrogen-bond geometry (Å, º)

**Table:**

*D*—H···*A*	*D*—H	H···*A*	*D*···*A*	*D*—H···*A*
O1—H1*OA*···Cl2^i^	0.82 (2)	2.37 (3)	3.107 (3)	150 (4)
O1—H1*OB*···Cl1^ii^	0.82 (2)	2.27 (2)	3.086 (3)	173 (4)
O2—H2*OA*···Cl9^iii^	0.82 (2)	2.33 (2)	3.120 (3)	161 (4)
O2—H2*OB*···Cl8^iv^	0.82 (2)	2.28 (2)	3.095 (3)	177 (4)
O3—H3*OA*···Cl5^iii^	0.85 (2)	2.30 (2)	3.100 (3)	158 (4)
O3—H3*OB*···Cl6^iv^	0.83 (2)	2.28 (2)	3.106 (3)	173 (4)

Symmetry codes: (i) −*x*+1/2, *y*+1/2, −*z*+3/2; (ii) −*x*+1/2, *y*−1/2, −*z*+3/2; (iii) −*x*+3/2, *y*+1/2, −*z*+3/2; (iv) −*x*+3/2, *y*−1/2, −*z*+3/2.
